# Factors influencing patients’ satisfaction at different levels of health facilities in Bangladesh: Results from patient exit interviews

**DOI:** 10.1371/journal.pone.0196643

**Published:** 2018-05-16

**Authors:** Gourab Adhikary, Md. Shajedur Rahman Shawon, Md. Wazed Ali, Md. Shamsuzzaman, Shahabuddin Ahmed, Katya A. Shackelford, Alexander Woldeab, Nurul Alam, Stephen S. Lim, Aubrey Levine, Emmanuela Gakidou, Md. Jasim Uddin

**Affiliations:** 1 Health Systems and Population Studies Division, icddr,b, Mohakhali, Dhaka, Bangladesh; 2 Cancer Epidemiology Unit, Nuffield Department of Population Health, University of Oxford, Oxford, United Kingdom; 3 Expanded Programme on Immunisation, DGHS, Mohakhali, Dhaka, Bangladesh; 4 Institute for Health Matrices and Evaluation (IHME), University of Washington, Seattle, Washington, United States of America; Yokohama City University, JAPAN

## Abstract

There is a paucity in current literature about the level of patients’ satisfaction and factors influencing it in Bangladesh health system. We aimed to measure the level of patients’ satisfaction across different types and levels of healthcare facilities and to determine which factors influence this satisfaction level. A patient exit interview was carried out among 2207 patients attending selected health facilities in two administrative divisions of Bangladesh, namely Rajshahi and Sylhet. Information on healthcare experience and satisfaction with received care was collected through an electronic structured questionnaire. Information about *‘overall satisfaction with healthcare’* was collected on a 10-point scale and then dichotomized based on the median-split. Binomial logistic regressions, both simple and multivariable, were conducted to identify which factors contribute significantly to patients’ satisfaction. We found that 63.2% of the participants were satisfied with the healthcare service they received. Patients attending the private facilities had the highest level of satisfaction (i.e. 73%) and patients attending the primary care facilities had the lowest level of satisfaction (i.e. 52%). Factors like convenient opening hours, asking related questions to the providers, facility cleanliness and privacy settings were significantly associated with patients’ satisfaction. Being satisfied with facility cleanliness (multivariable OR 4.30; 95% CI: 3.29–5.62) and privacy settings (multivariable OR 1.68; 95% CI: 1.28–2.21) were the strongest predictors of patients’ satisfaction. In conclusion, a significant portion of the patients in Bangladesh are not satisfied with their received care. Patients’ satisfaction can be increased by focusing on improving facility cleanliness, privacy settings and providers’ interpersonal skills.

## Introduction

Patients’ satisfaction reflects patients’ perceptions and needs towards health service utilization. Assessing patients’ satisfaction is important since it often helps, in absence of healthcare service quality indicators, to determine the quality of health-care delivery and health system responsiveness [1,2]. Higher levels of patients’ satisfaction indicate higher levels of patient empowerment, commitment to care and compliance to recommended management–all of which results in better health outcomes [3,4]. Measuring patients’ satisfaction also helps to improve service delivery and to prioritize capacity building needs and resource distribution [5,6]. Previous studies looked into this topic either from quality of care perspectives [7,8] or from healthcare system perspectives [9,10].

Factors that influence patients’ satisfaction with healthcare services can be classified into two broad categories: provider-related and patient-related [2,11–13]. Since healthcare service engages both healthcare providers and patients, it is crucial to measure patients’ satisfaction in relation to patients’ socioeconomic characteristics. A recent systematic review [11] found that providers’ competence, interpersonal skills and facility characteristics (e.g. physical environment, type and level of facility) were positively associated with patients’ satisfaction. Conversely, patient-related characteristics, for example, gender, age, race, socio-economic status, heath status and expectation were weak and inconsistent predictors of patients’ satisfaction. Several studies also highlighted how much patient’s perceptions of care and actual healthcare experiences contribute to overall patients’ satisfaction level [1,14].

The number of studies looking at patients’ satisfaction in low and middle-income countries is increasing [5,15–18]. In Bangladesh, patients’ satisfaction was studied focusing on maternal and neonatal health [19], family planning services [20], diabetes [21]. Aldana *et al*. [22] found that providers’ attitude towards the patients and reduced waiting time influenced patients’ satisfaction significantly in the rural public health facilities. But, no study has yet compared patients’ satisfaction level among different types and levels of healthcare facilities.

The health service delivery system in Bangladesh comprised of public, private and NGO facilities. The Ministry of Health and Family Welfare (MoHFW) plans and implements the public healthcare delivery through various healthcare infrastructure, from national to the community level. The organization of public healthcare facilities is as follows: super-specialized postgraduate teaching institutions at national level; medical college hospitals, infectious disease and general hospitals at divisional level; district hospital at district level; upazilla health complex (UHC) at upazilla (sub-district–a sub-unit of districts, a geographical region used for administrative purposes analogous to that of a borough of Western countries) level; union health and family welfare centers (UHFWC) and union sub-centers (USC) at union level; and community clinics (CC) at community level. Besides, private healthcare facilities, ranging from super-specialized hospitals to community level pharmacies, mushroomed in the last two decades. The government spent 3.5% of the gross domestic product (GDP) on health and most of the healthcare expenditure is from out-of-pocket [23].

Better understanding of the patients’ satisfaction with the healthcare delivery and determinants of it across different levels of healthcare facilities will help the stakeholders to develop and implement public health programs tailored to patients’ expectation and needs. In this paper, we aimed to measure the level of patients’ satisfaction across different types and levels of healthcare facilities and to determine which factors influence this satisfaction level.

## Methods

This study was approved by the institutional review board at University of Washington, Seattle, USA and International Centre for Diarrhoeal Disease Research, Bangladesh (icddr,b). All study participants provided informed written consent for their participation.

### Study design and sampling

This study is based on the patient exit interview survey, conducted between March and August in 2015, from the assessment of access, bottlenecks, costs and equity in health sector of Bangladesh (ABCE) study. The overall objective of ABCE study was to assess the constraints, costs, capacity and provision of service, quality of care, continuity, and demand-side constraints in healthcare delivery in private and public facilities in Bangladesh. This study had three components: i) health facility survey, ii) patient exit interview, and iii) District Civil Surgeon Office or City Corporation office survey.

This survey was conducted in two (out of seven) purposely selected divisions of Bangladesh based on their disparities in health indicators, namely Rajshahi as the high performing division and Sylhet as the low performing division. From Rajshahi division one rural area (e.g. Joypurhat district) and one urban area (e.g. Rajshahi City Corporation) were selected. From Sylhet division also, one rural area (e.g. Sylhet district) and one urban area (e.g. Sylhet City Corporation) were selected. The healthcare facilities were sampled from each selected districts and city corporations where the facilities with mix of public and private, different tiers, with variation in sizes and patient loads. Patient exit interviews were conducted at all sampled facilities.

The selected respondents were patients or caregivers, 18 years of age or older, and leaving the facility after attempting to receive healthcare services from the selected healthcare facility. We excluded respondents if they were under 18 years of age and without attendant aged 18 years of age or more and/or unable to answer the survey questions due to physical or mental condition preventing participation. Depending on the size of the facility and patient load, up to 30 patient exit interviews were conducted from each facility. We selected 2331 patients and asked them to participate in this study. Among them, 111 refused to participate, three did not fulfill the eligibility criteria and ten did not provide consent to participate yielding 2207 patients to be included in this study (final response rate 94.7%) [Table 1].

**Table 1:** Distribution of patients by facility type who participated in the patient exit survey.

Facility type	No. of patient surveyed	Percent
Medical College Hospital	159	7.2
District Sadar/General Hospital	63	2.9
Maternal and Child Welfare Centre (MCWC)	130	5.9
Upazila Health Complex (UHC)	258	11.7
Union Sub-center (USC)	104	4.7
Union Health and Family Welfare Centre (UHFWC)	202	9.2
Community Clinic (CC)	361	16.4
Private Hospital	213	9.7
Private Clinic	272	12.3
NGO Hospital	2	0.1
NGO clinic	115	5.2
Pharmacy	328	14.9
Total	2207	100.0

### Data collection

Data were collected through an electronic structured questionnaire. The research assistants interviewed the selected participants if they fulfilled the eligibility criteria. The exit interview collected information on participants’ sociodemographic characteristics, type of health service received, time and costs associated with the visit and perceived quality and satisfaction of services.

### Study variables

Patient’s perception regarding facility opening hours was collected with five responses: *“very inconvenient”*, *“somewhat inconvenient”*, *“neither inconvenient nor convenient”*, *“somewhat convenient”* and *“very convenient”*. We categorized the first three response categories into *“inconvenient”* and latter two response categories into *“convenient”* for analysis purpose. Questions regarding satisfaction with facility’s cleanliness and privacy settings had five responses–*“very bad”*, *“bad”*, *“moderate”*, *“good”* and *“very good”*. We categorized the first three responses as *“not satisfied”* and latter two as *“satisfied”*.

Information about the outcome variable, *‘overall satisfaction with healthcare’* was collected on a scale of 0 to 10 where 0 indicated no satisfaction at all and 10 indicated highest possible satisfaction with the healthcare received. The distribution of overall satisfaction is highly skewed to the left; so, we dichotomized overall satisfaction based on median-split into *“not satisfied (< 8)”* and *“satisfied (≥8)”*.

### Data analyses

We assessed the levels of patients’ satisfaction according to healthcare facility types (public and private) and levels (primary care, higher care, community clinics and pharmacies). We analyzed patients from community clinics and pharmacies separately (although they belong to the primary care level in Bangladesh health system) because of their obvious differences in structure and scope of services. Primary care facilities included Maternal and Child Welfare Centre (MCWC), Upazila Health Complex (UHC), Union Sub-center (USC) and Union Health and Family Welfare Centre (UHFWC). District hospitals, NGO clinics and hospitals, private clinics and hospitals and medical college hospitals were grouped into higher care facilities.

Participants’ sociodemographic characteristics are summarized and presented as percentages for categorical variables and as mean ± SD for continuous variables. We looked at the patients’ experience variables by overall and across various facility types and levels. We performed binomial logistic regression to identify the factors influencing patients’ satisfaction. We calculated both unadjusted and adjusted odds ratios (OR) with 95% confidence interval (95% CI). For adjusted analyses, we developed two models: a minimally-adjusted model in which we adjusted for patient's age, sex, area, division, household monthly income and health status; and a fully-adjusted model in which we additionally included all the explanatory variables simultaneously. All statistical analyses were conducted using Stata 14 version.

## Results

### Characteristics of patients

Majority of the participants were female (61%) and resided in rural areas (67%). There were more participants from Rajshahi division than from Sylhet division (53.2% vs. 46.8%). Almost half of the participants were between 18 and 39 years old. Islam was the major religion (94%) among the participants. One-fifth of the participants did not have any formal education whereas more than half of the participants had either primary or secondary education. Fifty-seven percent of the participants reported their occupation as homemaker. Mean number of household member was 5.4 ± 2.9 and mean monthly household income was 16,953 ± 28,541 Bangladeshi Taka [Table 2].

**Table 2:** Sociodemographic characteristics of the participants from the patient exit survey (n = 2207), presented as percentage or Mean ± SD.

Variables	Categories	Number	Percentage
Sex	Male	863	39.1
	Female	1343	60.9
Division	Rajshahi	1175	53.2
	Sylhet	1032	46.8
Area	Rural	1486	67.3
	Urban	721	32.7
Age	<18 years	393	17.9
	18–29 years	659	30.0
	30–39 years	396	18.0
	40–49 years	305	13.9
	50–59 years	214	9.8
	≥ 60 years	227	10.3
Religion	Islam	2080	94.4
	Hinduism	116	5.3
	Others	8	0.4
Education level	No education	351	19.8
	Primary education	461	26.0
	Secondary education	620	35.0
	Higher secondary education	167	9.4
	Bachelor degree or above	174	9.8
Occupation	Unemployed	336	17.1
	Homemaker	1121	57.1
	Semi-skilled/ skilled labor	221	11.3
	Businessman	244	12.4
	Professionals	41	2.1
Number of household members	Mean ± SD	5.4 ± 2.9	
Monthly household income (in BDT)	Mean ± SD	16,953 ± 28,541	
Marital status	Unmarried	181	13.3
	Widowed	23	1.7
	Divorced	7	0.5
	Married	1149	84.5

BDT: Bangladeshi Taka; SD: standard deviation

Number of missing cases: Sex = 0; division = 0; area = 0; age category = 13; religion = 3; education level = 434; occupation = 244; household member = 7; monthly income = 115; marital status = 847.

### Patient’s experiences in healthcare facilities

Table 3 and Table 4 show the distribution of various aspects of patient’s healthcare experience according to different types and levels of healthcare facilities. Community clinics and pharmacies were the two most accessible facilities in shortest time (i.e. less than 15 minutes). Conversely, only 17% respondents could travel to higher facilities within 15 minutes. Most of the respondents reported that the facility opening hours were convenient for them to visit, irrespective of facility levels and types. In all types of healthcare facilities except the higher care ones, more than half of the patients had to wait less than half an hour before getting treatment from the provider. The proportions of patients who were prescribed and given medicine directly were 96% and 91% for community clinics and pharmacies, respectively. The rates of providing prescription and drugs were less than 50% in other kinds of facilities. In the higher care facilities, almost one-third of the patients received care from more than one provider and around 90% received treatment from a doctor. In pharmacies, only 17% of the patients could ask questions about health problem to the provider whereas in the higher care facilities 61% could ask. Overall, most respondents were satisfied with cleanliness and privacy settings across all levels of healthcare facilities, but the levels of satisfaction in these aspects were higher in private and higher care facilities than the rest of healthcare facilities.

**Table 3:** Patients’ healthcare experiences according to facility types, presented as percentages.

Variables	Categories	Public facilities ^a^	Private facilities ^b^	Overall
Number of patients		1277	930	2207
Travel time to reach the facility	Under 15 mins	43.2	36.9	40.6
	16–29 mins	26.7	22.0	24.7
	30 mins to 1 hour	17.7	21.6	19.3
	1–2 hour	9.4	15.1	11.8
	More than 2 hours	3.0	4.4	3.6
Facility opening hours	Inconvenient	10.8	12.4	11.4
	Convenient	89.2	87.6	88.6
Waiting time in the facility	Less than 30 minutes	51.0	55.7	53.0
	30 minutes to 1 hour	31.6	27.2	29.8
	1 to 2 hours	10.0	11.3	10.5
	More than 2 hours	7.4	5.7	6.7
Medicines	Not prescribed	39.3	47.6	42.8
	Prescribed	4.3	6.5	5.2
	Prescribed & given directly	56.5	45.9	52.0
First visit to the facility as patient	Yes	10.7	27.2	17.7
	No	89.3	72.8	82.3
No. of providers providing care	One	83.9	75.9	80.5
	More than one	16.1	24.1	19.5
Provision of care involving a doctor	Yes	43.1	53.0	47.3
	No	56.9	47.0	52.7
Asking question about health problem to the provider	Yes	41.2	44.1	42.4
	No	58.8	55.9	57.6
Cleanliness of the facility	Good	66.8	80.7	72.4
	Bad	33.2	19.3	27.6
Privacy settings	Good	56.0	78.6	65.4
	Bad	44.0	21.4	34.6

^a^ Public facilities included community clinics, Union Health and Family Welfare Center (UHFWC), Union Sub-center (USC), Upazilla Health Complex (UHC), Medical College hospitals, District Sadar/General hospitals

^b^ Private facilities included private hospitals, private clinics, NGO clinics and pharmacies

**Table 4:** Patients’ healthcare experiences according to various levels of facilities, presented as percentages.

Variables	Categories	Primary care facilities ^a^	Higher care facilities ^b^	Pharmacies	Community clinics
Number of patients		694	707	328	361
Travel time to reach the facility	Under 15 mins	39.9	16.4	66.2	62.9
	16–29 mins	28.8	23.2	19.8	23.3
	30 mins to 1 hour	18.3	30.0	9.1	11.4
	1–2 hour	10.5	22.1	4.3	2.2
	More than 2 hours	2.4	8.3	0.6	0.3
Facility opening hours	Inconvenient	10.7	12.1	14.8	8.8
	Convenient	89.3	87.9	85.2	91.2
Waiting time in the facility	Less than 30 minutes	45.7	33.0	86.7	75.8
	30 minutes to 1 hour	33.9	34.5	12.0	24.2
	1 to 2 hours	10.2	21.9	0.6	0.0
	More than 2 hours	10.2	10.7	0.6	0.0
Prescription and medicine	Not prescribed	45.5	73.6	5.9	3.6
	Prescribed	6.6	7.1	3.1	0.8
	Prescribed & given drugs directly	47.9	19.3	91.0	95.5
First visit to the facility as patient	Yes	12.6	28.4	15.9	3.9
	No	87.4	71.6	84.1	96.1
No. of providers providing care	One	79.2	68.7	100.0	98.6
	More than one	20.8	31.3	0.0	1.4
Provision of care involving a doctor	Yes	50.4	89.1	0.0	0.6
	No	49.6	10.9	100.0	99.4
Asking question about health problem to the provider	Yes	34.8	61.2	17.1	37.7
	No	65.2	38.8	82.9	62.3
Cleanliness of the facility	Good	65.6	78.3	68.8	68.4
	Bad	34.4	21.7	31.2	31.6
Privacy settings	Good	58.7	83.6	55.4	42.0
	Bad	41.3	16.4	44.6	58.0

^a^ Primary care facilities included Union Health and Family Welfare Center (UHFWC), Union Sub-center (USC) and Upazilla Health Complex (UHC)

^b^ Higher care facilities included Medical College hospitals, District Sadar/General hospitals, private hospitals and private clinics

### Patients’ satisfaction level and factors influencing it

**Fig 1:**
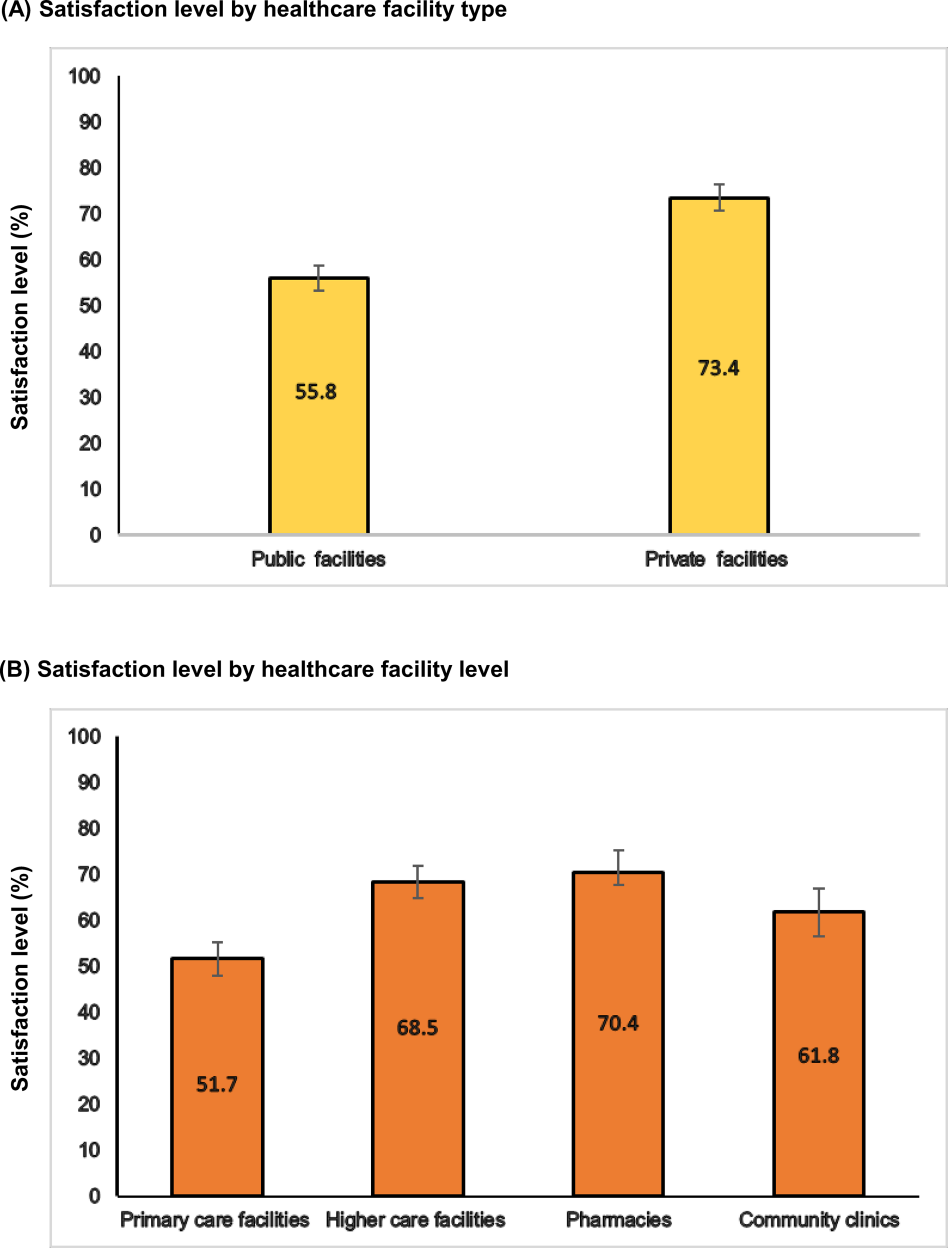
**Levels of patients’ satisfaction (%) with 95% confidence intervals according to (A) healthcare facility types and (B) healthcare facility levels.** Public facilities included community clinics, Union Health and Family Welfare Center (UHFWC), Union Sub-center (USC), Upazilla Health Complex (UHC), Medical College hospitals, District Sadar/General hospitals. Private facilities included private hospitals, private clinics, NGO clinics and pharmacies. Primary care facilities included Union Health and Family Welfare Center (UHFWC), Union Sub-center (USC) and Upazilla Health Complex (UHC). Higher care facilities included Medical College hospitals, District Sadar/General hospitals, private hospitals and private clinics.

Fig 1 shows the levels of patients’ satisfaction by overall and facility types and levels. Overall, 63.2% (95% CI: 61.2% - 65.3%) of all respondents were satisfied with their received care. Private facilities had the highest level of patients’ satisfaction (i.e. 73.4%; 95% CI: 70.6% - 76.3%). In primary care and public facilities, relatively less proportions of respondents were satisfied (i.e. 52% and 56%, respectively). However, pharmacies had relatively higher level of satisfaction i.e. 70.4% (95% CI: 65.5% - 75.4%).

Table 5 illustrates the results of simple and multivariate logistic regressions with levels of patients’ satisfaction as the dependent variable and user experiences as the predictor variables. Patients who received healthcare services in private facilities were more satisfied in compared with patients who received it in public facilities (multivariable OR 2.64; 95% CI: 1.61–4.32). Convenient opening hours and asking provider questions about health problem were significantly associated with patients’ satisfaction level. Being satisfied with facility cleanliness (multivariable OR 4.30; 95% CI: 3.29–5.62) and privacy settings (multivariable OR 1.68; 95% CI: 1.28–2.21) were the strongest predictors of patients’ satisfaction.

**Table 5:** Factors influencing patients’ satisfaction from logistic regression with odds ratio (OR) and 95% confidence intervals (CIs).

Variables	Categories	Unadjusted OR (95% CI)	Adjusted OR (95%CI)	
			Minimally-adjusted model ^a^	Fully- adjusted model ^b^
Facility type	Public	Reference	Reference	Reference
	Private	2.19 (1.82–2.63)	2.44 (1.67–3.57)	2.64 (1.61–4.32)
Travel time to reach the facility	Under 15 mins	Reference	Reference	Reference
	16–29 mins	0.8 (0.64–1.0)	0.85 (0.66–1.09)	0.97 (0.71–1.33)
	30 mins to 1 hour	0.83 (0.66–1.06)	0.84 (0.63–1.11)	0.86 (0.60–1.24)
	1–2 hour	0.83 (0.62–1.10)	0.83 (0.60–1.17)	0.67 (0.42–1.05)
	More than 2 hours	1.45 (0.86–2.43)	1.19 (0.67–2.12)	1.20 (0.56–2.57)
Facility opening hours	Inconvenient	Reference	Reference	Reference
	Convenient	1.10 (0.81–1.48)	1.50 (1.08–2.10)	1.64 (1.10–2.43)
Waiting time before getting treatment	Less than 30 minutes	Reference	Reference	Reference
	30 minutes to 1 hour	0.88 (0.72–1.08)	0.92 (0.73–1.16)	1.15 (0.85–1.53)
	1 to 2 hour	0.81 (0.61–1.09)	0.87 (0.61–1.22)	1.13 (0.72–1.76)
	More than 2 hours	0.79 (0.56–1.13)	0.89 (0.61–1.32)	0.88 (0.53–1.46)
Prescription and medicine	Not prescribed	Reference	Reference	Reference
	Prescribed	1.04 (0.69–1.56)	0.98 (0.64–1.53)	1.05 (0.56–2.01)
	Prescribed & given drugs directly	0.96 (0.8–1.15)	1.04 (0.81–1.33)	1.08 (0.76–1.52)
First visit to the facility as patient	No	Reference	Reference	Reference
	Yes	0.97 (0.77–1.21)	0.80 (0.62–1.05)	0.72 (0.49–1.07)
No. of providers providing care	One	Reference	Reference	Reference
	More than one	1.22 (0.97–1.52)	0.98 (0.75–1.29)	1.13 (0.78–1.65)
Provision of care involving MBBS doctor	No	Reference	Reference	Reference
	Yes	1.09 (0.91–1.29)	1.10 (0.84–1.46)	1.11 (0.73–1.69)
Asking provider questions about health problem	No	Reference	Reference	Reference
	Yes	1.51 (1.27–1.81)	1.40 (1.15–1.72)	1.39 (1.08–1.80)
Cleanliness of the facility	Bad	Reference	Reference	Reference
	Good	4.35 (3.53–5.35)	4.09 (3.27–5.12)	4.30 (3.29–5.62)
Privacy settings	Bad	Reference	Reference	Reference
	Good	2.77 (2.3–3.33)	2.68 (2.16–3.33)	1.68 (1.28–2.21)

^a^ Minimally-adjusted model was adjusted for facility level, patient's age, sex, area, division, household monthly income and health status

^b^ Fully-adjusted model was adjusted for all the explanatory variables simultaneously in addition to variables already adjusted in the minimally-adjusted model

## Discussion

In this study, we found that overall 63.2% patients were satisfied with the healthcare received in Bangladesh and the level of satisfaction varied by facility types and levels—in private facilities the satisfaction level was found to be the highest. Factors like convenient opening hours, asking provider questions about health problem, provision of better cleanliness and privacy settings were found to be significantly associated with patients’ satisfaction. We also explored the differences in patient experiences and satisfaction across different levels and types of healthcare facilities in Bangladesh. We found that while facilities like community clinics and pharmacies had lesser travel time, waiting time and higher availability of prescribed drugs, patients were relatively unsatisfied with their cleanliness and privacy settings. On the other hand, higher level facilities and private facilities despite longer travel time and waiting time, had better cleanliness, privacy settings and interpersonal communication with patients.

Our study found a striking difference in satisfaction level between public and private facilities (e.g. 56% vs. 73%). Previous studies also shows that the public health sector in Bangladesh suffers from perceived low quality of service [5,22]. Among the public-sector facilities, community clinics in the ward level had better satisfaction among patients than facilities in the union and upazila levels, 62% and 52%, respectively. Though community clinics have shortage of running water supply which is essential for cleanliness and sufficient space for maintaining privacy, they had lesser travel time and waiting time and around 96% of the patients got their prescribed medicines readily available in the facility. Moreover, the higher satisfaction level in private facilities might be related to their better cleanliness and privacy settings.

The proportion of patients satisfied with rendered care in our study (i.e. 63.2%) was somewhat similar to that of Aldana *et al*. (i.e. 68%). In addition, they reported that around half of the female patients were dissatisfied with the received services [22]. Islam *et al*. found relatively higher level (i.e. 85%) of satisfaction with maternal and neonatal health services [19]. In our multivariate analysis cleanliness of the facility was the strongest predictor of patients’ satisfaction, followed by satisfactory privacy settings, convenient opening hours and provision of asking questions to the providers. We did not find any association with travel time and waiting time. In contrary to our findings, a previous study in Bangladeshi context found providers’ politeness, respect for privacy, duration of consultation and waiting time strongly predicted patients’ satisfaction [22].

In our study, it was evident that when patients could ask questions to the healthcare providers regarding their diagnosis and management plan, they seemed to be more satisfied. Previous literature also suggested that healthcare providers’ interpersonal communication skills and behavior towards the patients were directly linked with patients’ satisfaction [11,24]. However, more than half of the surveyed patients could not ask questions to their providers. Previous study reported that only 31% of the patients could ask questions at the district hospitals whereas only 10% could do that in upazila health complexes [19]. Some people would argue that shortage of healthcare personnel in the facilities leads to overburdening of workload on the existing personnel affecting their ability and morale to render quality care. Shortage of healthcare personnel also compels the providers to shorten the consultation time and thus does not let patients ask questions to their providers. Previous study conducted in rural Bangladesh also revealed that average consultation time was very low (average 2.33 minutes) and only 49% patient had some sort of explanations regarding the their health problems [22]. Recent reviews on patients’ satisfaction also highlighted that importance of providers’ interpersonal communication skills outweighs their technical competence and recommended to strengthen training and evaluation on providers’ interpersonal skills and empathetic skills [2,11,24].

This study emphasized the importance of physical environment, such as cleanliness and maintaining adequate privacy to provide quality care. Physical environment of a health facility could potentially influence patients’ satisfaction. Previous studies in various contexts have shown that a convenient and comfortable facility environment leads to better patients’ satisfaction [5,11,15–18]. In Bangladeshi context, privacy of the clients is not always adequately maintained, especially for females. Aldana *et al*. reported only 45% patient who received care for maternal and/or family planning services were satisfied with privacy maintenance during consultation [22]. In our study, we found satisfaction with cleanliness was higher among patients attending higher-level facilities which contradicts with findings from Islam et al.–better cleanliness at upazila health complexes than district hospitals (82.5% vs. 50.0%) [19].

Measuring patients’ satisfaction has several purposes, for example–i) to assess quality of care [25]; ii) to compare various health interventions and/or systems [7]; iii) to identify gaps in service provision components and recommend interventions to improve [26]. However, though there has been a growing evidence on patients’ satisfaction as surrogate indictor of quality of care, patients’ satisfaction has remained an ambiguous and complex concept with numerous predictors [2]. Moreover, assessment of quality of care through the patients’ satisfaction lens is highly individualistic and may reflect only part of health system responsiveness. Hashting *et al*. argued that patients’ satisfaction studies may hinder the optimum care [12]. Bleich *et al*. reported that only 17.5% of total variance in patients’ satisfaction with health system was attributed to patient experience and related variables; indicating a considerable gap in our understanding of underlying factors contributing to patients’ satisfaction [1].

The present study is subject to several limitations. Since the underlying health facilities, from where patients were surveyed, were purposively selected, the representativeness of the findings may be limited. Moreover, observational study design, lack of appropriate control groups and residual confounding may alter the associations between predictors and patients’ satisfaction. We collected self-reported satisfaction from the patients which is highly subjective to social desirability bias, as patients might have given responses that would please healthcare providers instead of true reflection of their satisfaction. Additionally, we categorized satisfaction level based on median-split and patients who responded below the median may still have believed that they were satisfied with the services. Therefore, this approach might underestimate the proportion of patients who were satisfied with the care. The main strength of this study is large number of participants from various types and levels of health facilities. Moreover, we tried to control for a wide range of confounders in the multivariate model to identify possible determinants of patients’ satisfaction.

## Conclusion

In conclusion, a significant portion of the patients in Bangladesh are not satisfied with received care. Patients’ satisfaction can be improved by focusing on improving facility cleanliness, privacy settings and providers’ interpersonal skills. The results of this study could help stakeholders focus on specific dimensions of service provision to improve the overall quality of care.

## Supporting information

S1 File Blank questionnaire. (DOC)

S2 File Data set. (DTA)
