# Supplementary material for: Factors influencing patients’ satisfaction at different levels of health facilities in Bangladesh: Results from patient exit interviews
Source: PLoS One. 2018 May 16;13(5):e0196643. doi: 10.1371/journal.pone.0196643 (PMC5955531; doi:10.1371/journal.pone.0196643)
Supplement: S1 File — (DOC) [file pone.0196643.s001.doc]

**Survey Specifications**

**ABCE Patient Exit Interview**

**SURVEY Questionnaire**

**Color-Coding Legend**

Highlighted text = Programming notes

Highlighted text = Preloaded variable from participant list

**Reminder to interviewer:**

*All text in italics should be read aloud to interviewees by you.*

**All text in bold are directions for you and should not be read aloud.**

Normal text should not be read aloud, but can be used by you to prompt interviewees, as needed.

|  |  | | **Questionnaire Identifiers** |  |  |  |
| --- | --- | --- | --- | --- | --- | --- |
|  | FIELDSTAFF_ID | | What is your interviewer ID: : সাক্ষাত্কার গ্রহনকারীর আইডি ________________ | Dynamic Answers from Interivewer List |  |  |
|  | FACILITY_CAT | | Category of the facility: সেবা কেন্দ্রের ধরণ__ | Dynamic Answers from facility Category |  |  |
|  | FACILITY_TYPE | | Type of facility: সেবা কেন্দ্রের প্রকার_____ | Dynamic Answers from Facility List, filter by FACILITY_CAT |  |  |
|  | FACILITY_ID | | Facility ID: সেবা কেন্দ্রের আইডি _______ | Dynamic Answers from MP Facility Sample, filter by FACILITY_TYPE |  |  |
|  | WARD_CONFIRM | | Is this the correct Ward ওয়ার্ড আইডি নাম্বার লিখুন? [ward_id] |  |  |  |
|  | UNION_CONFIRM | | Is this the correct Union/Zone ইউনিয়ন/জোন আইডি নাম্বার লিখুন? [union_id] |  |  |  |
|  | UPAZILA_CONFIRM | | Is this the correct Upazila/Municipality উপজেলা/মিউনিসিপালিটি আইডি নাম্বার লিখুন? [union_id] |  |  |  |
|  | DISTRICT_CONFIRM | | Is this the correct District/City Corporation জেলা/সিটি কর্পোরেশন আইডি নাম্বার লিখুন? [district_id] |  |  |  |
|  | AUTO_ID | | AutoID Number: স্বয়ংক্রিয় আইডি নাম্বার: | Surveybe AutoID questionnaire identifier |  |  |
|  |  | | **Introduction ভূমিকা** |  |  |  |
|  | TIME_START | | **Survey Start Time: সাক্ষাৎকার শুরুর সময়** | Surveybe Time Stamp |  |  |
|  |  | | **Once you are positioned outside of the health facility, identify target patient interviewee and begin to determine if they meet eligibility criteria**  আপনার অবস্থান যদি স্বাস্থ্য সেবা কেন্দ্রের বাহিরে হয়, তাহলে রোগীর তথ্যদাতা খুজে বের করুন এবং তাদের উপযুক্ততার মানদন্ড পূরণ হচ্ছে কিনা তা যাচাই কারুন  **Once potential interviewee is sighted, begin recruitment script now**  **তাদের মধ্যে থেকে যে ভাল জানে তাকে আলা্দা করুন এবং সাক্ষাতকার প্রক্রিয়া শুরু করুন** |  |  |  |
|  | REFUSE | | Hello, my name is __________ and I am contacting you on behalf of International Centre for Diarrhoeal Disease Research, Bangladesh (icddr,b) to gather information on the demand for health services at this health facility. May I please ask you a few questions?  আমার নাম………. আমি আইসিডিডিআর,বি এর পক্ষ থেকে আপনার কাছে এসেছি এই সেবা কেন্দ্রের সেবা বিষয়ক কিছু তথ্য জানতে। আমি এই বিষয়ে আপনাকে কিছু প্রশ্ন করতে পারি?   - 1=Yes, continue to survey হ্যাঁ, সাক্ষাৎকার চলবে   0=Declines কথা বলতে নারাজ | Rout to COMMENTS if REFUSE = 0 |  |  |
|  |  | | **To clarify who is a patient and who is an attendant accompanying them, ask:** *Who came to this facility to receive medical services during this visit?*  কে রোগী এবং কে রোগীর সহযোগী তা নিশ্চিত হওয়ার জন্য তাদেরকে জিজ্ঞাসা করুন, তাদের মধ্যে কে চিকিৎসা সেবা নিতে এসেছেন?  **If there are multiple patients, assign each patient a number sequentially, starting at one. For example if there are three patients, assign them the numbers 1, 2, and 3.**  যদি অনেক জন একসাথে চিকিৎসা নিতে এসে থাকেন, প্রত্যেকের জন্য এক থেকে সিরিয়াল নম্বর প্রদান করুন।উদাহরণস্বরূপ যদি তিনজন রোগী থাকে, তাহলে তাদের জন্য নাম্বার হবে 1, 2 এবং 3। |  |  |  |
|  | SEX_PT | | Patient sex **(observed)** রোগী মহিলা না পুরুষ তা দেখে সিলেক্ট করুন)  **(SELECT ONE) (একটি উত্তর নির্বাচন করুন)**   - 1=Male পুরুষ - 2= Female মহিলা - 3=Transgender হিজরা |  |  |  |
|  | PT_AGE | | **Ask the patient:** How old are you? আপনার বয়স কত?  **Probe for complete age.** (পূর্ণ বয়স যাচাই করুণ)  **If the patient is a child (under the age of 18) they are not eligible to participate unless they are accompanied by an attendant that is 18 or older.**  যদি রোগী শিশু হয় (বয়স যদি 18 বছরের নিচে হয়) তাহলে তিনি এ গবেষণায় বয়স বিবেচনায় অংশগ্রহণ করার উপযুক্ত নন, যদি না তার সাথের সহযোগীর বয়স 18 বছর বা তার বেশী হয় ।  **If the patient is a child or is unable to respond for any reason and they are accompanied by an attendant, ask the attendant:**  যদি রোগী শিশু হয় বা কোন কারণে তথ্য দিতে অপরাগ হন তাহলে তার সাথে যদি সহযোগী থাকে তবে তাকে জিজ্ঞাসা করুন  How old is the patient? রোগীর বয়স কত?  **Enter 0 if the patient has not yet turned 1.** যদি রোগীর বয়স এখনও 1 বছর পূর্ণ না হয়, তাহলে 0 লিখুন ।  **Response Codes উত্তর কোড**  Enter -1 for Don’t Know জানি না  Enter -2 for Decline to respond উত্তর দিতে নারাজ  Age (patient) turned at last birthday:  শেষ জন্মদিনে রোগীর বয়স কত হয়েছে____ |  |  |  |
|  | PT_AGE_CAT | | Could you tell me the age range if I read the different options to you?  আমি কিছু বয়সের রেন্জ বলি এর মধ্যে রোগীর বয়সের রেন্জ কোনটি দয়া করে বলুন?  **(SELECT ONE) (একটি উত্তর** নির্বাচন করুন)  **Choose what is most appropriate.** সবচেয়ে কাছাকাছি **উত্তরটি নির্বাচন করুন**   - 1- Under 5 (5 বৎসরের নিচে) - 2- 5-9 (5 থেকে 9 বৎসরের মধ্যে) - 3- 10-19 (10 থেকে 19 বৎসরের মধ্যে) - 4- 20-29 (20 থেকে 29 বৎসরের মধ্যে) - 5- 30-39 (30 থেকে 39 বৎসরের মধ্যে) - 6- 40-49 (40 থেকে 49 বৎসরের মধ্যে) - 7- 50-59 (50 থেকে 59 বৎসরের মধ্যে) - 8- 60-69 (60 থেকে 69 বৎসরের মধ্যে) - 9- 70+ (70 বা এর বেশী) - -1=Don’t know *জানি না* - -2=Decline to respond *উত্তর দিতে নারাজ* | IF PT_AGE=-1 OR -2 |  |  |
|  | PT_16 | | *Are you less than 18 years of age? আপনার বয়স কি* 18 **বছরের নিচে?**   - 1- Yes হ্যাঁ - 2- No না - -1- Don’t know জানিনা - -2- Decline to respond উত্তর দিতে নারাজ | IF  PT_AGE_CAT=-1, -2 |  |  |
|  | ELIGIBLE1 | | **If the patient is under 18 and unaccompanied by an attendant you must end the survey.**  **If no attendant is present, tell the respondent:** *Unfortunately you are ineligible to participate in our survey, but thank you for taking the time to answer the questions you did.*  *যদি রোগী* বয়স 18 *বৎসরের নিচে হয় এবং তার সাথে কেউ না থাকে তাহলে সাক্ষাৎকার এখানেই শেষ করতে হবে । যদি কোন সহযোগী না থাকে তাহলে উত্তরদাতাকে বলেন:* বয়স বিবেচনায় আমরা আপনার স্বাক্ষাৎকার নিতে পারছি না, *সময় দিয়ে প্রশ্নের উত্তর দেওয়ার জন্য আপনাকে ধন্যবাদ ।*  **(SELECT ONE) (একটি উত্তর** নির্বাচন করুন)   - 2- Continue, attendant(s) is present, *সহযোগী* উপস্থিত থাকলে স্বাক্ষাৎকার চালিয়ে যান - 1 - End survey due to patient ineligibility রোগীর বয়স 18 এর নিচে হওয়ায় সাক্ষাৎকার গ্রহণকরা সম্ভব হলো না | Enable if PT_AGE < 18 OR PT_AGE_CAT = 1 OR PT_18 IN (1,-1,-2)  Rout to COMMENT if ELIGIBLE1=1 |  |  |
|  | RESP_PT | | **Identify respondent below.**  নিচে উত্তরদাতা নির্বাচন করুন.  **The attendant should answer the survey questions if the patient has cognitive or other impairments preventing him/her from answering questions** সহযোগীকে প্রশ্নের উত্তর দিতে হবে যদি রোগী প্রশ্নের উত্তর দিতে জ্ঞানীয় বা অন্যান্য ইন্দ্রিয়গুলোর জন্য বাধাপ্রাপ্ত হন ।    **Attendants must be 18 or older.** সহযোগী অবশ্যই 18 বা তদুর্ধ্ব হতে হবে.  **(SELECT ONE) (একটি উত্তর** নির্বাচন করুন)   - 1- Patient রোগী - 0- Attendant সহযোগী - 2-End survey, no attendant present কোন সহযোগী না থাকলে সাক্ষাৎকার শেষ করুন |  |  |  |
|  |  | | **If there are multiple attendants ask:** *Who spent the most time with the patient during your visit to this facility? এই সেবাগ্রহনের সময় যদি* একাধিক সহযোগী থাকে, তাহলে তাদেরকে জিজ্ঞাসা করুন রোগীর সাথে সেবাকেন্দ্রে কে বেশী সময় ব্যয় করেন**?**  **Make sure the person identified is 18 or older. If not, select another attendant. Once this person has identified themselves, say:**  নির্বাচিত ব্যক্তির বয়স 18 বা তার বেশী কিনা নিশ্চিত করুন । যদি না হয়, অন্য সহযোগী নির্বাচন করুন । আন্যদের থেকে একজনকে নির্বাচন করুন।  *I would like to ask you a few questions regarding the care received by this patient at this facility during this visit.* আমি আপনাকে সেবাকেন্দ্র পরিদর্শনের সময় রোগী কোন কোন সেবা পেয়েছে সেসম্পর্কে কিছু প্রশ্ন জিজ্ঞাসা করবো। | Enable if RESP_PT = 0 |  |  |
|  | AT_SEX | | Attendant sex **(observed)** সহযোগীর লিঙ্গ (পর্যবেক্ষণ করে লিখুন)  **(SELECT ONE) (একটি উত্তর নির্বাচন করুন)**   - 1- Male ছেলে - 2- Female মেয়ে - 3- Transgenger- হিজরা | Enable if RESP_PT = 0 |  |  |
|  | AT_AGE | | *How old are you* **(the attendant)***?*  **Probe for complete age, If the attendant is under 16, the survey will end. আপনার (সহযোগী) বয়স কত? পূর্ণ বয়স যাচাই করুন, যদি সহযোগীর** বয়স **18 বছরের নিচে হয়, তাহলে সাক্ষাতকার এখানেই শেষ** করুন **।**  **Response Codes উত্তর কোড**  Enter -1 for Don’t Know –জানিনা  Enter -2 for Decline to respond উত্তর দিতে নারাজ  Enter age in years বয়স **পূর্ণ বছরে লিখুন** | Enable if RESP_PT = 0 |  |  |
|  | AT_AGE_CAT | | *Could you tell me the age range if I read the different options to you?*  আমি কিছু বয়সের রেন্জ বলি এর মধ্যে আপনার বয়সের রেন্জ কোনটি দয়া করে বলুন?  **(SELECT ONE) একটি উত্তর নির্বাচন করুন**  **Choose what is most appropriate সঠিক উত্তরটি নির্বাচন করুন**   - *1- Under 18 (18* বছরের নিচে *)* - *3- 19-29 (19* থেকে 29 বৎসরের মধ্যে) - *4- 30-39 (30* থেকে 39 বৎসরের মধ্যে) - *5- 40-49 (40* থেকে 49 বৎসরের মধ্যে) - *6- 50-59 (50* থেকে 59 বৎসরের মধ্যে) - *7- 60-69 (60* থেকে 69 বৎসরের মধ্যে) - *8- 70+* (70 বা এর বেশী) - -1- Don’t know জানিনা - -2- Decline to respond উত্তর দিতে নারাজ | Enable if RESP_PT = 0  AND IF AT_AGE=-1 ,-1,-2 |  |  |
|  | AT_16 | | *Are you less than 18 years of age?* **(The attendant) আপনার (**সহযোগী**) বয়স কি** 18 বৎসরের **নিচে?**   - 1- Yes হ্যাঁ - 2- No না - -1- Don’t know জানিনা - -2- Decline to respond উত্তর দিতে নারাজ | Enable if RESP_PT = 0  IF AT_AGE_CAT=-1 OR -2 |  |  |
|  | ELIGIBLE2 | | **If the attendant is under 18, answers doesn’t know or declines to respond to their age, he/she is ineligible to be the respondent for this survey; select another attendant who has spent time with the patient during their visit at the health facility, if another attendant is available.**  **If no other attendant is present end the survey and tell them:** *Unfortunately you are ineligible to participate in our survey, but thank you for taking the time to answer the questions you did***.**  **যদি সহযোগী 18 বছরের নিচে হয়** এবং **উত্তর জানি না বা উত্তর দিতে নারাজ হয় তাহলে সে সাক্ষাৎকারের জন্য উপযুক্ত নয়; যদি** আরো **সহযোগী থাকে** তাহলে **তাদের থেকে একজন সহযোগী নির্বাচন করুন যে** সেবাগ্রহণের সময় রোগীর সাথে ছিলো **।** *যদি কোন সহযোগী না থাকে তাহলে সাক্ষাৎকার এখানেই শেষ করুন এবং* তাকে *বলুন:* আপনি *সাক্ষাৎকার দেওয়ার জন্য* উপযুক্ত নন*, তবুও সময় দিয়ে প্রশ্নের উত্তর দেওয়ার জন্য আপনাকে ধন্যবাদ ।*  **(SELECT ONE) (একটি উত্তর নির্বাচন করুন)**   - 1 Continue, attendant(s) is *সহযোগী* উপস্থিত থাকলে সাক্ষাৎকার চালিয়ে যান - 0 - End survey due to patient ineligibility রোগী বা সহযোগী সাক্ষাৎকারের জন্য অনুপযুক্ত হলে সাক্ষাৎকার এখানেই শেষ করুন | Enable if AT_AGE< 18 OR AT_AGE_CAT = 1 OR  AT_18 = 1 |  |  |
|  | CONSENT | | **OBTAIN CONSENT**  সম্মতি গ্রহন  *Instructions to interviewer: Please provide explanation about our study and give/read consent form and obtain consent.* *সাক্ষাৎকার* গ্রহণকারীর নির্দেশনাঃ গবেষণার বিষয় সম্পর্কে ব্যাখ্যা দিন এবং সম্মতি পত্র পড়তে দিন বা পড়ে শোনান ও সম্মতি নিন ।  *Do you consent to participate in this study?* আপনি কি এ গবেষণায় অংশগ্রহন করতে রাজি আছেন?  Consent Obtained? সম্মতি দিয়েছে?  **(SELECT ONE) (একটি উত্তর নির্বাচন করুন)**   - 1- Yes হাঁ - 0- No না | Enable if (PT_AGE > 15 OR PT_AGE_CAT >1 OR PT_18 = 0) OR (AT_AGE >15 ORAT_AGE_CAT> 1 OR AT_18 = 0)  Rout to COMMENT if CONSENT = 0 |  |  |
|  | PQ_MEDTX | | *Did you/the patient come to this facility to get medical consultation/treatment or for some other reason?* আপনি বা রোগী কি সেবাকেন্দ্রে চিকিৎসা পরামর্শ/সেবা বা অন্য কোন কিছু পাওয়ার জন্য এসেছিলেন?  **(SELECT ONE) (একটি উত্তর নির্বাচন করুন)**   - 0- Medical consultation/vaccination/treatment চিকিৎসা পরামর্শ / টিকাদান / চিকিৎসা - 1- Other reason,specify অন্য কারণ, নির্দিষ্ট করুন - -1- Don’t know জানিনা - -2- Decline to respond উত্তর দিতে নারাজ | Rout to COMMENT if PQ_MEDTX=1, -1 OR -2. |  |  |
|  |  | *The next questions I am going to ask are about the time and costs associated with getting to the facility.* পরবর্তী প্রশ্নে আমি আপনাকে সেবা কেন্দ্র সম্পর্কিত সময় এবং খরচ নিয়ে জিজ্ঞেস করবো? | |  |  |  |
|  | T_TIME | | *How long did it take you to travel to this facility today/this visit?* এই সেবা গ্রহণের জন্য আজ আপনার সেবাকেন্দ্রে আসতে কত সময় লেগেছে?  **(Choose appropriate range for response) (সঠিক রেঞ্জটি নির্বাচন করুন)**  **(SELECT ONE) (একটি উত্তর নির্বাচন করুন)**   - 1- Under 15 minutes 15 মিনিটের নিচে - 2- 16-29 minutes 16 থেকে 29 মিনিটের মধ্যে - 3- 30 minutes-1 hour 30 থেকে 1 ঘন্টার মধ্যে - 4- 1-2 hours 1 থেকে 2 ঘন্টার মধ্যে - 5- 2-3 hours 2 থেকে 3 ঘন্টার মধ্যে - 6- Over 3 hours 3 ঘন্টার বেশী - -1- Don’t know জানি না - -2- Decline to respond উত্তর দিতে নারাজ | EITHER RESPONDENT |  |  |
|  | T_TIMERATE | | *Would you say this travel time is: very long, long, reasonable, short or very short?*  আপনি যাতায়াতের সময়টা বলুন: খুব বেশী, বেশী, সন্তোসজনক, অল্প নাকি খুব অল্প?  **(SELECT ONE) (একটি উত্তর নির্বাচন করুন)**   - *1- Very long* খুব বেশী - *2- Long* বেশী - *3- Reasonable* সন্তোসজনক - *4- Short* অল্প - *5- Very short* খুব অল্প - -1- Cannot say বলতে পারি না - -2- Decline to respond উত্তর দিতে নারাজ | EITHER RESPONDENT |  |  |
|  | T_MODE | | *What mode of transportation did you use to come to this facility today/this visit?* আপনি আজকে এই সেবাকেন্দ্রে আসার সময় কোন ধরনের যানবাহন ব্যবহার করেছেন?  **(SELECT ALL THAT APPLY) (একটি উত্তর নির্বাচন করুন)**   - 1- Walked হেটেঁ - 2- Rickshaw/Paddle-Van রিক্সা বা ভ্যান - 3- Autorickshaw/CNG অটোরিক্সা বা সিএনজি - 4- Private vehicle (for example, own car or taxi) ব্যক্তিগত যানবাহন (উদাহরণ: নিজের গাড়ি বা ট্যাক্সি) - 5- Ambulance এম্বুলেন্স - 6- Bus বাস - 7- Train ট্রেন - 8- Bicycle বাইসাইকেল - 9- Others (Specify) অন্যান্য (নির্দিষ্ট করুন) - -1- Don’t know জানিনা - -2- Decline to respond উত্তর দিতে নারাজ |  |  |  |
|  | T_MODE_OSP | | *Please describe other mode of transportation:* (যানবাহনের ধরণ বর্ণনা করুন) |  |  |  |
|  | T_COST | | *How much will be the total cost of transportation for this visit? Include the cost (to and from) for the patient and any accompanying individuals.* (এই সেবাগ্রহণের জন্য যাতায়াত খরচ কত লাগবে? রোগী এবং রোগীর সহযোগীর প্রত্যেকের খরচ (আসা-যাওয়া) আলাদাভাবে অন্তর্ভূক্ত করুন  **Response code (উত্তর কোড)**   - Enter -1 for Don’t Know জানি না - Enter -2 for Decline to respond উত্তর দিতে নারাজ | EITHER RESPONDENT  IF T_MODE=1,2,5,6,7,8 |  |  |
|  | T_COSTRATE | | *How would you rate the cost of transportation?* আপনি যাতাযাত খরচ কিভাবে মূল্যায়ন করবেন?  **(SELECT ONE) (একটি উত্তর নির্বাচন করুন)**   - *1- Very expensive* খুব ব্যয়বহুল - *2- Somewhat expensive* কিছুটা ব্যয়বহুল - *3- Neither inexpensive nor expensive* সস্তাও না ব্যয়বহুলও না - *4- Somewhat inexpensive* কিছুটা সস্তা - *5- Very inexpensive or free* অত্যন্ত সস্তা বা বিনামূল্যে - -1- Cannot say বলতে পারে না - -2- Decline to respond উত্তর দিতে নারাজ | EITHER RESPONDENT  Show if T_COST>0 |  |  |
|  | HRS_KNOW | | *Do you know what hours this facility is open?*  আপনি কি জানেন সেবা কেন্দ্র কখন খুলে?  **(SELECT ONE) (একটি উত্তর নির্বাচন করুন)**   - 1- Yes হ্যাঁ - 2- No না - -1- Don’t know জানি না - -2- Decline to respond উত্তর দিতে নারাজ | EITHER RESPONDENT |  |  |
|  | HRS_RATE | | *How would you rate the hours this facility is open?*  আপনি কিভাবে সেবা কেন্দ্র খোলার সময়টা মূল্যায়ন করবেন?  **(SELECT ONE) (একটি উত্তর নির্বাচন করুন)**   - *1- Very Inconvenient* অত্যন্ত অসুবিধাজনক - *2-Somewhat Inconvenient* কিছুটা অসুবিধাজনক - *3- Neither inconvenient nor* convenient সুবিধাজনকও না অসুবিধাজনকও না - *4- Somewhat convenient* কিছুটা সুবিধাজনক - *5- Very convenient* অত্যন্ত সুবিধাজনক - -1- Cannot say বলতে পারি না - -2- Decline to respond উত্তর দিতে নারাজ | EITHER RESPONDENT  Enable if HRS_KNOW=1 |  |  |
|  |  | | *The next questions I’m going to ask are about the costs and ease of receiving medical attention and medicines at this facility.* (পরবর্তী প্রশ্নে আমি আপনাকে সেবা কেন্দ্রের গ্রহণকৃত প্রতিটি সেবা ও ওষুধবাবদ খরচ নিয়ে জিজ্ঞাসা করবো ) |  |  |  |
|  | W_TIME | | *How long did you/the* ***patien****t wait between arriving at the facility and receiving medical attention in this visit from any medical personnel? If no medical services were received today, how long did you wait until leavingthe facility?* আপনি বা রোগী সেবা কেন্দ্রে পৌছার পর চিকিৎসা কর্মীর কাছ থেকে সেবা পাওয়ার জন্য কতক্ষণ অপেক্ষা করতে হয়েছিলো? আপনি যদি আজকে কোন সেবা না নিয়ে থাকেন তাহলে সেবাকেন্দ্রে কতক্ষণ অপেক্ষা করতে হয়েছিলো?  **(SELECT ONE) (একটি উত্তর নির্বাচন করুন)**   - 1- Under 30 minutes 30 মিনিটের নিচে - 2- 30 minutes-1 hour 30 মিনিট থেকে1 ঘন্টার মধ্যে - 3- 1-2 hours 1 ঘন্টা থেকে 2 ঘন্টার মধ্যে - 4- 2-3 hours 2 ঘন্টা থেকে 3 ঘন্টার মধ্যে - 5- Over 3 hours 3 ঘন্টার বেশী - -1- Don’t know জানি না - -2- Decline to respond উত্তর দিতে নারাজ |  |  |  |
|  | W_TIMERATE | | *How would you rate this length of time you waited before receiving medical attention or before leaving the facility without receiving medical attention?* আপনি সেবা গ্রহণের পূর্বের অপেক্ষার সময়টাকে বা সেবাকেন্দ্রে সেবা না গ্রহণ করার দিনকার অপেক্ষার সময়টাকে কিভাবে মূল্যায়ন করবেন?  **(SELECT ONE) (একটি উত্তর নির্বাচন করুন)**   - *1- Very long* খুব বেশী - *2- Long* বেশী - *3- Reasonable* সন্তোসজনক - *4- Short* অল্প - *5- Very short* খুব অল্প - -1- Cannot say বলতে পারি না - -2- Decline to respond উত্তর দিতে নারাজ | EITHER RESPONDENT  IF W_TIME=1,2,3 OR 4 |  |  |
|  | PAY | | *During this visit did you/the patient pay any fees that realted to the treatment other than tips? This includes payments for medicines, tests, procedures, medical consultations, registration or patient card feesand any other medical services or supplies. এই সেবাগ্রহনের সময়* আপনি বা রোগীকে টিপস ছাড়া চিকিৎসাবাবদ কোন ধরনের ফি দিতে হয়েছিলো? ওষুধ, পরীক্ষা, পদ্ধতি, চিকিৎসা পরামর্শ, রেজিস্ট্রেশন বা রোগীর কার্ড ফি এবং আন্যান্য চিকিৎসা সেবা বা সরবরাহের জন্য টাকা দিতে হয়েছিলো  **(SELECT ONE)** **(একটি উত্তর নির্বাচন করুন)**   - 1- Yes হ্যাঁ - 2- No না - -1- Don’t know জানি না - -2- Decline to respond উত্তর দিতে নারাজ |  |  |  |
|  | CARD | | *What was the fee you paid for the patient ticket/book or registration, if any?* আপনাকে রোগীর টিকেট/বই বা রেজিস্ট্রেশনবাবদ কত টাকা দিতে হয়েছিলো?  **Response Code (উত্তর কোড)**  Enter -1 for Don’t Know জানি না  Enter -2 for Decline to respond উত্তর দিতে নারাজ  Enter -3 for Not applicable (no fee for patient card or book) প্রজোয্য নয় (রোগীর কার্ড বা বই বাবদ কোন খরচ হয়নি | Enable if PAY=1 |  |  |
|  | CARDRATE | | *How would you rate the cost of patient ticket/book or registration?* আপনি রোগীর টিকেট/বই বা রেজিস্ট্রেশনবাবদ খরচটাকে কিভাবে মূল্যায়ন করবেন?  **(SELECT ONE)**  **(একটি উত্তর নির্বাচন করুন)**   - *1- Very expensive* অত্যন্ত ব্যয়বহুল - *2- Somewhat expensive* কিছুটা ব্যয়বহুল - *3- Neither inexpensive nor expensive* মোটামুটি - *4- Somewhat inexpensive* কিছুটা কম - *5- Very inexpensive or free* অত্যন্ত সস্তা বা বিনামূল্যে - -1- Cannot say বলতে পারি না - -2- Decline to respond উত্তর দিতে নারাজ | Enable if CARD>0 |  |  |
|  | USERFEE | | *What was the total cost of medical consultation for this visit? এই সেবাগ্রহনের* জন্য চিকিৎসা পরামর্শবাবদ মোট কত খরচ হয়েছে?  **Response code (উত্তর কোড)**  Enter -1 for Don’t Know জানি না  Enter -2 Decline to respond উত্তর দিতে নারাজ  Enter -3 for Not applicable (Consultation or user fee did not apply for this visit) প্রজোয্য নয় (*এই সেবাগ্রহনের* জন্য পরামর্শ বা ব্যবহার ফি প্রজোয্য নয়) | Enable if PAY=1 |  |  |
|  | USERFEE_RATE | | *How would you rate the cost of medical consultation?* আপনি *এই* চিকিৎসা *সেবাগ্রহনের* খরচটাকে কিভাবে মূল্যায়ন করবেন?  **(SELECT ONE) (একটি উত্তর নির্বাচন করুন)**   - *1- Very expensive* (অত্যন্ত ব্যয়বহুল) - *2- Somewhat expensive* (কিছুটা ব্যয়বহুল) - *3- Neither inexpensive nor expensive* মোটামুটি - *4- Somewhat inexpensive* কিছুটা সস্তা - *5- Very inexpensive or free* অত্যন্ত সস্তা বা বিনামূল্যে - -1- Cannot say বলতে পারি না - -2- Decline to respond উত্তর দিতে নারাজ | Enable if USERFEE>0 |  |  |
|  | MEDS | | Were you/patient prescribed or given any medicines during this visit? *এই সেবাগ্রহনের* সময় কি কোন ওষুধ দেওয়া হয়েছে?  **(SELECT ONE) (একটি উত্তর নির্বাচন করুন)**   - 1= Yes, prescribed হ্যাঁ, ব্যবস্থাপত্র দিয়েছে - 2= Not prescribed ব্যবস্থাপত্র দেয় নাই - 3=Yes, given drugs directly হ্যাঁ, সরাসরি ওষুধ দিয়েছে - -1- Don’t know জানি না - -2- Decline to respond উত্তর দিতে নারাজ | EITHER RESPONDENT |  |  |
|  | M_GOT | | *Were you able to get all of the medicines you needed from this facility, some of them, or none?* আপনার প্রয়োজনীয় সব ওষুধ কি সেবাকেন্দ্র থেকে পেয়েছেন বা কিছু পেয়েছেন নাকি একটিও না?  **(SELECT ONE) (একটি উত্তর নির্বাচন করুন)**   - 1-Did not try to get medicines from this facility সেবাকেন্দ্র থেকে ওষুধ পাওয়ার চেষ্টা করা হয় নাই - 2- None একটাও না - 3- Some of them কিছু - 4- Yes, All of them হ্যাঁ, সবগুলো - -1- Don’t know জানি না - -2- Decline to respond উত্তর দিতে নারাজ | Enable if MEDS =1 or 4  EITHER RESPONDENT |  |  |
|  | M_GOTWHY | | *Why were you unable to obtain the medicines from this facility?* আপনি সেবাকেন্দ্র থেকে ওষুধ নিতে পারেন নাই কেন?  **(SELECT ONE) (একটি উত্তর নির্বাচন করুন)**   - 1- Too expensive খুবই ব্যয়বহুল - 2- Medicine(s) not in stock স্টকে ওষুধ ছিলো না - 3- Referred elsewhere by doctor ডাক্তার অন্যত্র রেফার করেছে - 4- Requested for outside prescription for some medicines বাহির থেকে ওষুধ কিনতে অনুরোধ করেছে - 5- Responsible person absent দায়িত্বপ্রাপ্ত ব্যক্তি অনুপস্থিত ছিলো - 6- Other (specify) অন্যান্য (নির্দিষ্ট করুন) - -1- Don’t know জানি না - -2- Decline to respond উত্তর দিতে নারাজ | Enable if M_GOT<4  EITHER RESPONDENT |  |  |
|  | M_COST | | *What was the total cost of medicines for this visit? এই সেবাগ্রহনের* সময় ওষুধের জন্য মোট কত খরচ হয়েছিলো?  **Response codes (উত্তর কোড)**  Enter 0 if Free বিনামূল্যে  Enter -1 if Don’t Know জানি না  Enter -2 if Decline to respond উত্তর দিতে নারাজ | Enable if MEDS = 3 or 4 AND if PAY = 1  EITHER RESPONDENT |  |  |
|  | M_COSTRATE | | *How would you rate the cost of medicines?* আপনি ওষুধবাবদ খরচকে কিভাবে মূল্যায়ন করবেন?  **(SELECT ONE) (একটি উত্তর নির্বাচন করুন)**   - *1- Very expensive* অত্যন্ত ব্যয়বহুল - *2- Somewhat expensive* কিছুটা ব্যয়বহুল - *3- Neither inexpensive nor expensive* মোটামুটি - *4- Somewhat inexpensive* কিছুটা সস্তা - *5- Very inexpensive or free* অত্যন্ত সস্তা বা বিনামূল্যে - -1- Cannot say বলতে পারে না - -2- Decline to respond উত্তর দিতে নারাজ | Enable if M_COST>0  EITHER RESPONDENT |  |  |
|  | T_COSTHOWMUCH | | *What was the total cost of tests and procedures for this visit? এই সেবাগ্রহনের* সময় পরীক্ষা এবং প্রক্রিয়াবাবদ মোট কত খরচ হয়েছে?  **Response codes (উত্তর কোড)**  Enter 0 if patient had tests and/or procedures that were free বিনামূল্যে পরীক্ষা এবং প্রক্রিয়া করা হয়েছে  Enter -1 if Don’t Know জানি না  Enter -2 if Decline to respond উত্তর দিতে নারাজ  Enter -3 if Did not have any tests or procedures কোন পরীক্ষা বা প্রক্রিয়া দরকার হয় নাই | Enable if PAY=1  EITHER RESPONDENT |  |  |
|  | PAY_ALL | | *Did you pay for all tests or procedures done today at this facility today, or only for some of them?* আপনি আজ এই সেবাকেন্দ্র থেকে সবগুলো পেয়েছেন কিনা?  **(SELECT ONE) (একটি উত্তর নির্বাচন করুন)**   - 1- None না - 2- Some of them কিছু - 3- Yes, All of them হ্যাঁ, সবগুলো - -1- Don’t know জানি না - -2- Decline to respond উত্তর দিতে নারাজ | Enable if T_COSTHOWMUCH>0  EITHER RESPONDENT |  |  |
|  | T_COSTHOWMUCHRATE | | *How would you rate the cost of tests and procedures?* আপনি পরীক্ষা এবং প্রক্রিয়া বাবদ খরচকে কিভাবে মূল্যায়ন করবেন?  **(SELECT ONE) (একটি উত্তর নির্বাচন করুন)**   - *1- Very expensive* অত্যন্ত ব্যয়বহুল - *2- Somewhat expensive* কিছুটা ব্যয়বহুল - *3- Neither inexpensive nor expensive*  মোটামুটি - *4- Somewhat inexpensive* কিছুটা সস্তা - *5- Very inexpensive or free* অত্যন্ত সস্তা বা বিনামূল্যে - -1- Cannot say বলতে পারে না - -2- Decline to respond উত্তর দিতে নারাজ | Enable if PAY=1 & T_COSTHOWMUCH>0  EITHER RESPONDENT |  |  |
|  | SUPPLIES | | *Did you have to purchase and/or provide any medical supplies (such as syringes, bandage, nebulizer,etc) for your treatment during this visit? If so, where did you purchase them?* আপনাকে কি চিকিৎসার জন্য *এই সেবাগ্রহনের* সময় কোন চিকিৎসা সরঞ্জামাদি কিনতে এবং বা প্রদান করতে হয়েছে (যেমন-সিরিঞ্জ,ব্যান্ডেজ, নেবুলাইজার, ইত্যাদি)? যদি তাই হয়, আপনি এগুলো কোথা থেকে কিনেছেন?  **(SELECT ONE) (একটি উত্তর নির্বাচন করুন)**   - *1- Yes, purchased from somewhere outside the facility* হ্যাঁ, সেবাকেন্দ্রের বাহির থেকে কিনেছে - *2- Yes, purchased from this facility* হ্যাঁ, এই সেবাকেন্দ্র থেকে কিনেছে - *3- Not needed* প্রয়োজন হয় নাই - *0- No* না - -1- Don’t know জানি না - -2- Decline to respond উত্তর দিতে নারাজ | EITHER RESPONDENT |  |  |
|  | S_COST | | *What was the total cost of these medical supplies?* চিকিৎসা সরঞ্জামবাবদ মোট কত খরচ হয়েছে?  **Response codes (উত্তর কোড)**  Enter -1 if Don’t Know জানি না  Enter -2 if Decline to respond উত্তর দিতে নারাজ | Enable if SUPPLIES=1 or 2  EITHER RESPONDENT |  |  |
|  | S_COSTRATE | | *How would you rate the cost of these medical supplies?* আপনি এই চিকিৎসা সরঞ্জামবাবদ খরচকে কিভাবে মূল্যায়ন করবেন?  **(SELECT ONE)(একটি উত্তর নির্বাচন করুন)**     - *1- Very expensive* অত্যন্ত ব্যয়বহুল - *2- Somewhat expensive* কিছুটা ব্যয়বহুল - *3- Neither inexpensive nor expensive* সস্তাও না ব্যয়বহুলও না - *4- Somewhat inexpensive* কিছুটা সস্তা - *5- Very inexpensive or free* অত্যন্ত সস্তা বা বিনামূল্যে - -1- Cannot say বলতে পারি না - -2- Decline to respond উত্তর দিতে নারাজ | Enable if S_COST>0  EITHER RESPONDENT |  |  |
|  | O_PAY | | *Did you make any other payments for health services or supplies during this visit?* আপনি এই সেবাগ্রহনের সময় স্বাস্থ্য সেবা বা চিকিৎসা সরঞ্জাম পাওয়ার জন্য অন্য কোন অর্থ প্রদান করেছেন?   - 1=Yes হ্যাঁ - 0- No না - -1- Don’t know জানিনা - -2- Decline to respond উত্তর দিতে নারাজ | enable if PAY=1  EITHER RESPONDENT |  |  |
|  | O_PAYTEXTT | | *What was the payment for? কিসের জন্য দিয়েছিলেন?*  FREE TEXT   - -1- Don’t know জানিনা - -2- Decline to respond উত্তর দিতে নারাজ | Enable if O_PAY= 1  EITHER RESPONDENT |  |  |
|  | O_COST | | *How much did you pay? কত দিয়েছিলেন?*  **Response codes**  Enter -1 for Don’t Know জানিনা  Enter -2 for Decline to respond উত্তর দিতে নারাজ | Enable if O_PAY=1  EITHER RESPONDENT |  |  |
|  | I_COST | | *What was the total cost of tips for this visit? এই সেবাগ্রহনের সময় সর্বমোট কত টাকা বকসিস দিয়েছিলেন?*  **Response codes**  Enter -1 if Don’t Know জানিনা  Enter -2 if Decline to respond উত্তর দিতে নারাজ  Enter -3 if NA (did not pay any tips) প্রযোজ্য নয়(কোন বকসিস দেইনি) | EITHER RESPONDENT |  |  |
|  | I_COSTRATE | | *How would you rate the cost of tips?* আপনি এই *বকসিস* বাবদ খরচকে কিভাবে মূল্যায়ন করবেন?  **(SELECT ONE)(একটি উত্তর নির্বাচন করুন)**   - *1- Very expensive* অত্যন্ত ব্যয়বহুল - *2- Somewhat expensive* কিছুটা ব্যয়বহুল - *3- Neither inexpensive nor expensive* সস্তাও না ব্যয়বহুলও না - *4- Somewhat inexpensive* কিছুটা সস্তা - *5- Very inexpensive or free* অত্যন্ত সস্তা বা বিনামূল্যে - -1- Cannot say বলতে পারি না - -2- Decline to respond উত্তর দিতে নারাজ | Enable if I_COST>0  EITHER RESPONDENT |  |  |
|  |  | | **PERCEIVED QUALITY OF SERVICES: মানসম্মত সেবাগ্রহন সম্পর্কিতঃ** | EITHER RESPONDENT |  |  |
|  | TY_VISIT | | Is this your/the patient’s first visit to this facility as a patient? *এটা কি* রোগী হিসাবে এই সেবাকেন্দ্রে আপনার/রোগীর প্রথম সেবাগ্রহন?  **(SELECT ONE)(একটি উত্তর নির্বাচন করুন)**   - 1- Yes হ্যাঁ - 0- No না - -1- Don’t know জানিনা - -2- Decline to respond উত্তর দিতে নারাজ |  |  |  |
|  | PQ_FIRST | | *Before this visit, have you ever been to this facility for any reason? This could include visiting a patient or accompanying a sick friend.*এই সেবাগ্রহনের আগে, আপনি কি কখনো কোন কারণে এই সেবা সেবাকেন্দ্রে এসেছিলেন ? কোন রোগী দেখতে বা কোন অসুস্থ্য বন্ধুর সহগামী হিসেবে এলেও তা অন্তর্ভুক্ত হবে।  **(SELECT ONE)(একটি উত্তর নির্বাচন করুন)**   - 1- Yes হ্যাঁ - 0- No না | EITHER RESPONDENT  Enable If TY_VISIT=1 |  |  |
|  |  | | ***Note to Interviewers :*** *সাক্ষাতকার গ্রহণকারীর জন্য নির্দেশনাঃ*  ***For “first time visit patients”*** *- For the next questions, I’d like you to think about when you decided to come to this facility for this visit as a patient.* প্রথমবার সেবাগ্রহনকারী রোগীদের জন্য" - পরের প্রশ্ন,আমি আপনার কাছে জানতে চাই , রোগী হিসাবে এই সেবাগ্রহনের জন্য কখন এই সেবাকেন্দ্রে আসার সিদ্ধান্ত নিয়েছিলেন?  ***For “follow-up patients”*** *- For the next questions, I’d like you to think about when you decided to come to this facility for the first time as a patient ফলো-আপের রোগীদের জন্য-পরের প্রশ্ন, আমি আপনার কাছে জানতে চাই, রোগী হিসেবে প্রথমবার সেবাগ্রহনের জন্য* কখন এই সেবাকেন্দ্রে আসার সিদ্ধান্ত নিয়েছিলেন? |  |  |  |
|  | PQ_REASON | | *What was the single most important reason you decided to come to this facility today?* আজ এই সেবাকেন্দ্রে আসার সিদ্ধান্ত নেয়ার ক্ষেত্রে সবচেয়ে গুরুত্বপূর্ণ কারণ কী ছিল? **Open ended prompt** (উন্মুক্ত প্রশ্ন, প্রম্পট)  **(SELECT ONE)(একটি উত্তর নির্বাচন করুন)**   - 1- Convenient location- সুবিধাজনক অবস্থান - 2- Convenient hours সুবিধাজনক সময় - 3- This facility is free এই সেবাকেন্দ্রে বিনামূল্যে সেবা দেয় - 4- Seeing a doctor is inexpensive কম খরচে ডাক্তার দেখানো যায় - 5- Medicines are inexpensive সস্তায় ঔষধ পাওয়া যায় - 6- Availability of medicines is good ঔষধ প্রাপ্যতা ভাল - 7- Good reputation of medical staff চিকিৎসা কর্মীদের ভাল সুনাম আছে - 8- Referred from another facility অন্য সেবাকেন্দ্রে রেফার হতে - 9- Advised by a health worker in the community to come here কমিউনিটি স্বাস্থ্য কর্মীর উপদেশ নিতে এখানে এসেছি - 10- Advised by family or friends to come here পরিবার বা বন্ধুদের পরামর্শে এখানে এসেছি - 11- It is the only facility available  এটাই একমাত্র সেবাকেন্দ্র - 12- My relative works here আমার আত্মীয় এখানে কাজ করে - 13- Others (Specify) অন্যান্য ( উল্লেখ করুন ) - -1- Don’t know জানিনা - -2- Decline to respond উত্তর দিতে নারাজ | EITHER RESPONDENT |  |  |
|  | PQ_REFERRED | | *Were/Was you/the patient referred to this facility or advised to come here by any of the following? আপনাকে/রোগীকে নিন্মলিখিত কেউ কি এই সেবাকেন্দ্রে আসার জন্য পরামর্শ দিয়েছিল?*  **(SELECT ALL THAT APPLY) (একাধিক উত্তর গ্রহনযোগ্য)**   - *1-* Health provider at another facility অন্য সেবাকেন্দ্রের সাস্থ্যসেবা প্রদানকারী - 2- Health worker in the community কমিউনিটি সাস্থ্য কর্মী - 3- Family or friend পরিবার বা বন্ধু - 4- Other (Specify) অন্যান্য ( বর্ণনা করুন) - 0- No না - -1- Don’t know জানিনা - -2- Decline to respond উত্তর দিতে নারাজ | EITHER RESPONDENT |  |  |
|  | PQ_REFERRED_OSP | | *Please describe other: অন্যান্য, দয়াকরে বর্ণনা করুনঃ* | Enable if Other selected in PQ_REFERRED |  |  |
|  | PQ_OVERALL | | *Before coming to the facility for the first time as a patient, what did you think of the overall quality of the facility? রোগী হিসেবে প্রথমবার এই সেবাকেন্দ্রে আসার আগে, এই সেবাকেন্দ্রের সার্বিক মান সম্পর্কে আপনার ধারনা কি ছিল?*  **(SELECT ONE)(একটি উত্তর নির্বাচন করুন)**   - *1- Very Bad- খুব খারাপ* - *2- Bad- খারাপ* - *3- Moderate মোটামুটি* - *4- Good ভাল* - *5- Very Good খুব ভাল* - -1- Cannot say বলতে পারিনা - -2- Decline to respond উত্তর দিতে নারাজ | EITHER RESPONDENT |  |  |
|  | PQ_WAIT | | *Before coming to this facility for the first time as a patient, how long did you expect the waiting time to be? রোগী হিসেবে প্রথমবার এই সেবাকেন্দ্রে আসার আগে, আপনি কত সময় অপেক্ষা করতে হবে বলে আশা করেছিলেন?*  **(SELECT ONE)(একটি উত্তর নির্বাচন করুন)**   - *1-Very long* অত্যন্ত দীর্ঘ সময় - *2- Long*  দীর্ঘ সময় - *3- Reasonable* যুক্তিসঙ্গত সময় - *4- Short* সংক্ষিপ্ত সময় - *5- Very short* অত্যন্ত সংক্ষিপ্ত সময় - -1- Cannot say বলতে পারিনা - -2- Decline to respond উত্তর দিতে নারাজ | EITHER RESPONDENT |  |  |
|  | PQ_EQUIP | | *Before coming to this facility for the first time as a patient, what did you think of the availability of medical equipment? রোগী হিসেবে প্রথমবার এই সেবাকেন্দ্রে আসার আগে,* চিকিৎসা সরঞ্জামাদির প্রাপ্যতা সম্পর্কে আপনি কি মনে করেছিলেন?    **(SELECT ONE)(একটি উত্তর নির্বাচন করুন)**   - *1- Very Bad খুব খারাপ* - *2- Bad খারাপ* - *3- Moderate মোটামুটি* - *4- Good- ভাল* - *5- Very Good খুব ভাল* - -1- Cannot say বলতে পারিনা - -2- Decline to respond উত্তর দিতে নারাজ | EITHER RESPONDENT |  |  |
|  | PQ_STAFF | | *Before coming to this facility for the first time as a patient, what did you think of the medical staff? রোগী হিসেবে প্রথমবার এই সেবাকেন্দ্রে আসার আগে, আপনি চিকিৎসা* কর্মী*দের সম্পর্কে কি মনে করেছিলেন?*  **(SELECT ONE)(একটি উত্তর নির্বাচন করুন)**   - *1- Very Bad খুব খারাপ* - *2- Bad খারাপ* - *3- Moderate মোটামুটি* - *4- Good- ভাল* - *5- Very Good খুব ভাল* - -1- Cannot say বলতে পারিনা - -2- Decline to respond উত্তর দিতে নারাজ | EITHER RESPONDENT |  |  |
|  | PQ_COST | | *Before coming to this facility for the first time as a patient, how expensive did you expect (your/the patient’s) treatment to be? রোগী হিসেবে প্রথমবার এই সেবাকেন্দ্রে আসার আগে,আপনি কেমন খরচ (আপনার/রোগীর) হবে বলে আশা করেছিলেন?*  **(SELECT ONE)(একটি উত্তর নির্বাচন করুন)**   - *Very expensive*  অত্যন্ত ব্যয়বহুল - *2- Somewhat expensive*  কিছুটা ব্যয়বহুল - *3- Neither inexpensive nor expensive*  সস্তাও না ব্যয়বহুলও না - *4- Somewhat inexpensive*  কিছুটা সস্তা - *5- Very inexpensive or free* অত্যন্ত সস্তা বা বিনামূল্যে - -1- Cannot say বলতে পারিনা - -2- Decline to respond উত্তর দিতে নারাজ |  |  |  |

|  | PQ_MEDS | *Before coming to this facility for the first time as a patient, how easy did you think it would be to obtain medicines? রোগী হিসেবে প্রথমবার এই সেবাকেন্দ্রে আসার আগে, কতটা সহজে ঔষধপত্র পাবেন বলে মনে করেছিলেন?*  **(SELECT ONE)(একটি উত্তর নির্বাচন করুন)**   - *1-Very Difficult* অত্যন্ত কঠিন - *2- Somewhat Difficult* কিছুটা কঠিন - *3- Neither difficult nor easy* কঠিনও না সহজও না - *4- Somewhat Easy* কিছুটা সহজ - *5- Very Easy* অত্যন্ত সহজ - -1- Cannot say বলতে পারি না - -2- Decline to respond উত্তর দিতে নারাজ | EITHER RESPONDENT |  |  |
| --- | --- | --- | --- | --- | --- |
|  | F_REASON_VAC | *Did you bring the child today for vaccination? আপনি কি আজ শিশুকে টিকা দিতে নিয়ে এসেছেন?*  *SELECT ONE* **(একটি উত্তর নির্বাচন করুন)**   - 1- Yes হ্যাঁ - 0- No না - -1- Don’t know জানিনা - -2- Decline to respond উত্তর দিতে নারাজ | ENABLE if PT_AGE<2 |  |  |
|  | VACRECEIVED | *Did your child receive vaccination today? আপনার শিশু কি আজ টিকা পেয়েছে?*  *SELECT ONE***(একটি উত্তর নির্বাচন করুন)**   - 1- Yes- হ্যাঁ - 0- No না - -1- Don’t know জানিনা - -2- Decline to respond উত্তর দিতে নারাজ | ENABLE if F_REASON_VAC=1 |  |  |
|  | NOVAC_WHY | *You said that you and the patient came to receive vaccines but the services were not received. Why did (you/the patient) not receive vaccines during this visit?*  আপনি বলেন যে, আপনি এবং রোগী টিকা নিতে এসেছিলেন কিন্তু টিকা নিতে পারেননি। কেন সেই ভিজিটের সময় টিকা নিতে পারেননি?  (SELECT ALL THAT APPLY)**(একাধিক উত্তর গ্রহনযোগ্য)**   - 1- Provider not available সেবাদানকারীকে পাওয়া যায়নি - 2- Vaccines were out of stock টিকা ছিল না - 3- Referred elsewhere অন্যত্র পাঠিয়েছিল - 4- Wait was too long অনেকক্ষণ অপেক্ষা করেছিলাম - 5- Clinic was closed ক্লিনিক বন্ধ ছিল - 6- Child was sick so vaccinator didn’t give vaccine শিশু অসুস্থ্য থাকায় টিকাদান কর্মী টিকা দেয়নি - 7- Child was outside the age range শিশু বয়সসীমার বাইরে ছিল - 7- Refused treatment সেবা দিতে নারাজ - 8- Staff was on strike স্টাফ ধর্মঘট ছিল - 9- Payment was too expensive ব্যয়বহুল ছিল - 10- Fear of side effects পার্শ্বপ্রতিক্রিয়ার ভয় - 11-Other reason, please describe below অন্য কারণ , নীচে বর্ণনা করুন - -1- Don’t know জানিনা - -2- Decline to respond - উত্তর দিতে নারাজ | Enable-If: F_RECEIVED=0 & F_REASON_VAC=1 |  |  |
|  | NOVAC_WHY_OSP | *Please describe other reason: অন্য কারন থাকলে দয়া করে বর্ণনা করুনঃ* | Enable if: NOVAC_WHY = 11 |  |  |
|  | *VAC_WHICH* | *Which vaccines did the patient receive today? You can give the name of the vaccine if you know it or the place on the child’s body where he/she got the vaccine. রোগী আজকে কি কি টিকা পেয়েছে? আপনি কি টিকাগুলোর নাম বলতে পারেন, যদি বলতে পারেন তাহলে বলুন শিশুদের শরীরের কোথায় কোথায় টিকা পেয়েছিল।*  **Do not read responses aloud. If respondent says a location on the body, mark this exactly as he or she describes it. উত্তরগুলো পড়ে শোনানো যাবে না।যদি উত্তরদাতা শরীরের কোন জায়গায় টিকা দিয়েছে তা সঠিকভাবে বলতে পারে সেগুলো কোড করুন।**  **(SELECT ALL THAT APPLY)(একাধিক উত্তর গ্রহনযোগ্য)**   - 1=BCG বিসিজি - 2=Penta পেনটা - 3= PCV পিসিবি - 4= Polio পোলিও - 5= MR এমআর - 6= Measles2^nd^ dose মিজেলস দ্বিতীয় ডোজ - 7= Drops in the mouth মুখের মধ্যে ফোটা - 8= A shot in the right leg ডান পায়ে দিয়েছিল - 9= A shot in the left leg বাম পায়ে দিয়েছিল - 10= A shot in the left arm বাম হাতে দিয়েছিল - -1- Don’t know জানিনা - -2- Decline to respond উত্তর দিতে নারাজ | Enable-If: F_RECEIVED=1 & F_REASON_VAC=1 |  |  |
|  | F_RECEIVED | *Did (you/the patient) receive health services this visit? Health services can include consultation, counseling, tests or procedures or receiving medicines. এইবার আপনি/রোগী কি সাস্থ্যসেবা গ্রহন করেছেন?*  স্বাস্থ্য পরিষেবা, পরামর্শ, পরীক্ষা নিরীক্ষা বা ওষুধ গ্রহণ পদ্ধতি সম্পর্কে জানা এর অন্তর্ভুক্ত হবে।  **(SELECT ONE)(একটি উত্তর নির্বাচন করুন)**   - 1- Yes হ্যাঁ - 0- No না - -1- Don’t know জানিনা - -2- Decline to respond উত্তর দিতে নারাজ | ENABLE if F_REASON_VAC=(0, -1, -2, .) |  |  |
|  | F_RECEIVED_WHY | *Why did (you/the patient) not receive health services this visit? এইবার আপনি/ রোগী কেন সাস্থ্যসেবা গ্রহন করেননি?*  **(f_re)**   - 1- Provider not available সেবাদানকারী ছিলনা - 2- Medical equipment not working চিকিৎসা সরঞ্জাম অকেজো ছিল - 3- Referred elsewhere অন্যত্র রেফার করেছে - 4- Wait was too long দীর্ঘ সময় অপেক্ষা করতে হয়েছিল - 5- Clinic was closed ক্লিনিক বন্ধ ছিল - 6- Medicines were out of stock স্টকে ওষুধ ছিল না - 7- Refused treatment চিকিৎসা দিতে নারাজ - 8- Staff was on strike স্টাফ ধর্মঘট ছিল - 9- Payment was too expensive খরচ অনেক বেশী - 10-Other reason, please describe below অন্য কারণ , নীচে বর্ণনা করুন - -1- Don’t know জানিনা - -2- Decline to respond উত্তর দিতে নারাজ | ENABLE if F_REASON_VAC=(0, -1, -2, .) |  |  |
|  | F_RECEIVED_WHY_OSP | *Please describe the other reason why (you/the patient) did not receive health services this visit? এইবার কেন আপনি সেবা গ্রহন করেননি তার অন্যান্য কারন থাকলে দয়া কারে বর্ণনা করুন?* | Enable-if Other was checked in F_RECEIVED_WHY |  |  |
|  |  | **Personnel and overall satisfaction**  ব্যক্তিগত এবং সার্বিক সন্তুষ্টি  ***Now I want to ask about your experience at this facility during this visit***  এখন আমি আপনার এই সেবাকেন্দ্রের আজকের অভিজ্ঞতা সম্পর্কে জানতে চাই।  *In the next questions I am going to ask are about how you felt about your experience at this facility during this visit. It will help if you can be honest because we want to know how services can be improved as well as what you thought was good.*  আমি পরের প্রশ্নগুলোতে জিজ্ঞাসা করতে যাচ্ছি, আপনি এই সেবাকেন্দ্রে সেবাগ্রহনের সময় আপনার কি অভিজ্ঞতা অনুভূত হয়েছে. আমরা জানতে চাই, কিভাবে সেবার মান আর উন্নত করা যায়, আপনি যেভাবে ভাল মনে করেন, সেবা ভাল ছিল কি, কিভাবে আরও উন্নত করা যায় জানতে চাই, আপনার পরামরশ সেবার মান উন্নত করতে সাহায্য করবে.  **Do not ever read the options** Don’t Know, Decline to Respond **or** Not Applicable*,* **only fill those in when indicated by the respondent.**  কখনও উল্লেখিত বিষয়গুলো পড়ে শুনাবেন না, শুধুমাত্র উত্তর জানি না, *উত্তর দিতে* নারাজ অথবা প্রযোজ্য না হলে তখন উত্তরদাতাকে বলুন। |  |  |  |
|  | PROVIDER_NO | *During your visit, from how many providers did you/the patient receive medical advice or treatment?*  আপননি/রোগী আজকে কতজন সেবাপ্রদানকারীর কাছ থেকে ডাক্তারী পরামর্শ বা চিকিৎসা পেয়েছেন?  **(SELECT ONE)** (একটি উত্তর নির্বাচন করুন)   - 11- One provider only শুধুমাত্র একজন সেবাপ্রদানকারীর কাছ থেকে - 2- More than one provider একের অধিক সেবাপ্রদানকারীর কাছ থেকে - -1- Don’t know জানিনা - -2- Decline to respond *উত্তর দিতে* নারাজ |  |  |  |
|  | P_PROVIDER | *During your visit, which of the following did you/the patient receive medical advice or treatment from?*  আজকে আপনি/রোগী নিম্নলিখিত কার কাছে পরামর্শ বা চিকিত্সা গ্রহন করেছেন?  **(SELECT ALL THE APPLY)**  একাধিক উত্তর গ্রহণযোগ্য   - *1*= MBBS Doctor এমবিবিএস ডাক্তার - *2=*SACMO (Sub Assistant Community Medical Officer)/Medical Assistantস্যাকমো/এমএ - *3=* FWV (Family welfare Visitor*)* এফডব্লিউভি - 4= Nurse/Paramedics নার্স/প্যারামেডিকস - 5= CHCP (Community Health Care Provider*)* সিএইচসিপি - 6= HA (Health Assistant*)* এইচএ - 7= FWA (Family Welfare Assistant*)* এফডব্লিউএ - 8=Counsellor কাউন্সেলর - 9= Pharmacist ফার্মাসিস্ট - 10= Other (Specify*)* অন্যান্য (বর্ণনা করুন) - -1- Don’t know জানি না - -2- Decline to respond উত্তর দিতে নারাজ |  |  |  |
|  | P_PROVIDER_RATE | *How would you rate your experience of being treated respectfully by the following personnel?*  *পূর্বে যে সব চিকিৎসা কর্মীদের কথা আলোচনা করা হলো তারা* আপনাকে যতটা সম্মানের সাথে সেবা দিয়েছেন সে সম্পর্কে আপনার মুল্যায়ন কি?  **(SELECT ONE)**(একটি উত্তর নির্বাচন করুন)   - 1- Very Bad খুব খারাপ - 2- Bad খারাপ - 3- Moderate মোটামুটি - 4- Good ভাল - 5- Very Good খুব ভাল - -1- Cannot say বলতে পারি না - -2- Decline to respond উত্তর দিতে সম্মত নন |  |  |  |
|  | RESPECT_NONMED | How would you rate your experience of being treated respectfully by the facility’s non-medical staff, such as those working at recept  এই সেবাকেন্দ্রের নন মেডিকেল স্টাফরা (যেমনঃ অভ্যর্থনায় কাজ করেন যারা তাদেরমত স্টাফ) যতটা সম্মানের সাথে সেবা দিয়েছেন তাকে আপনি কিভাবে মূল্যায়ন করবেন?  **(SELECT ONE)**(একটি উত্তর নির্বাচন করুন)   - 1- Very Bad খুব খারাপ - 2- Bad খারাপ - 3- Moderate মোটমিুটি - 4- Good ভাল - 5- Very Good খুব ভাল - -1- Cannot say বলতে পারি না - -2- Decline to respondউত্তর দিতে নারাজ | EITHER RESPONDENT |  |  |
|  | CLEAN | *How would you rate the cleanliness of the rooms inside this facility, including toilets if applicable?*  *এই সেবাকেন্দ্রের কক্ষের ভিতরের পরিস্কার-পরিচ্ছন্নতা যদি পায়খানা দেখে থাকেন তাসহ পরিস্কার-পরিচ্ছন্নতার মান সম্পর্কে আপনার মূল্যায়ন কি?*  **(SELECT ONE)** (একটি উত্তর নির্বাচন করুন)   - 1- Very Bad খুব খারাপ - 2- Bad খারাপ - 3- Moderate মোটামুটি - 4- Good ভাল - 5- Very Good খুব ভাল - -1- Cannot say বলতে পারি না - -2- Decline to respond উত্তর দিতে নারাজ | EITHER RESPONDENT |  |  |
|  | PRIVACY | *How would you rate the way your/the patients privacy was respected during physical examinations and treatments?*  আপনার/রোগীর শারীরিক পরীক্ষা এবং চিকিত্সার সময় যেভাবে গোপনীয়তা বজায় রেখেছিলেন সেটা আপনি কিভাবে মুল্যায়ন করবেন?  **(SELECT ONE)** (একটি উত্তর নির্বাচন করুন)   - 1- Very Bad খুব খারাপ - 2- Bad খারাপ - 3- Moderate মোটামুটি - 4- Good ভাল - 5- Very Good খুব ভাল - -1- Cannot say বলতে পারি না - -2- Decline to respond উত্তর দিতে নারাজ |  |  |  |
|  | TOPICS | *During the vaccination of your child, what information was provided by the health worker?*  আপনার সন্তানের টিকাদানের সময়, স্বাস্থ্য কর্মী কি কি তথ্য আপনাকে জানিয়েছিলেন?  **(SELECT ALL THAT APPLY**)(একাধিক উত্তর গ্রহনযোগ্য)   - - 0=Nothing was said কিছুই বলেনি   - 1= What injections were given on that day ঐ দিন কি ইঞ্জেকশন দিয়েছে তা বলেছেন   - 2= Diseases prevented by the injections given ইঞ্জেকশন দ্বারা কিকি রোগ প্রতিরোধ করা   - 3= Side effects of the vaccination and their management  টিকার পার্শ্বপ্রতিক্রিয়া ও তার ব্যবস্থাপনা   - 4= When to come for next vaccination পরবর্তী টিকার জন্য কখন আসতে হবে   - 5= Advised to hold on to card কার্ড যত্ন করে রাখতে বলেছে   - 6= Remind to bring card during the next visit পরবর্তী টিকার সময় কার্ড সঙ্গে আনতে বলেছে   - 7= Received health educational materials on vaccination and vaccination for reading পড়ার জন্য টিকা এবং টিকা উপর গৃহীত স্বাস্থ্য শিক্ষা উপকরণ দিয়েছে   - 8 = Other অন্যান্য   - -1=Don’t know জানিনা   - -2=Decline to respond *উত্তর দিতে* নারাজ | Enable if F_RECEIVED=1 & REASON_VAC=1 |  |  |
|  | EXPLAIN | *How would you rate your experience of how clearly explained things to you?*  *সেবাপ্রদানকারী আপনাকে বিষয়টি যেভাবে ব্যাখ্যা করেছেন* তাকে আপনি কিভাবে মুল্যায়ন করবেন?  *( SEPARATE QUESTION FOR EACH RESPONSE FROM MED_PER)*  **(SELECT ONE)** (একটি উত্তর নির্বাচন করুন)   - 1- Very Bad *অত্যন্ত খারাপ* - 2- Bad *খারাপ* - 3- Moderate *মোটামুটি* - 4- Good *ভাল* - *5-* Very Good *খুব ভাল* - *0-* No questions asked কোন প্রশ্ন জিজ্ঞাসা করি নাই - *-1-* Cannot say *বলতে পারিনা* - *-2-* Decline to respond *উত্তর দিতে* নারাজ | Separate question for each response from  EITHER RESPONDENT |  |  |
|  | QUESTIONS | *How would you rate your experience of getting enough time to ask questions about your/the patient’s health problem or treatment with (SEPARATE QUESTION FOR EACH RESPONSE FROM MED_PER)?* আপনার/রোগীর স্বাস্থ্য সমস্যা বা চিকিত্সা সম্পর্কে প্রশ্ন জিজ্ঞাসা করার জন্য যে সময় পেয়েছেন তাকে আপনি কিভাবে মুল্যায়ন করবেন?  *(SEPARATE QUESTION FOR EACH RESPONSE FROM MED_PER)*  **(SELECT ONE)**(একটি উত্তর নির্বাচন করুন)   - 1- Very Bad *অত্যন্ত খারাপ* - 2- Bad *খারাপ* - 3- Moderate *মোটামুটি* - 4- Good *ভাল* - *5-* Very Good *খুব ভাল* - *0-* No questions asked কোন প্রশ্ন জিজ্ঞাসা করি নাই - *-1-* Cannot say *বলতে পারিনা* - *-2-* Decline to respond *উত্তর দিতে* নারাজ | Separate question for each response from  EITHER RESPONDENT |  |  |
|  | ROOM_SPACE | *How would you rate the amount of space in the waiting and examination rooms?*  এই সেবা কেন্দ্রে অপেক্ষার জন্য এবং পরীক্ষা-নিরীক্ষার জন্য যে ব্যবস্থা আছে এটাকে আপনি কীভাবে মুল্যায়ন করবেন?  **(SELECT ONE)** (একটি উত্তর নির্বাচন করুন)   - 1- Very large *অত্যন্ত বড়* - 2- Large *বড়* - 3- Moderate *মোটামুটি* - 4- Small *ছোট* - 5- Very small *খুব ছোট* - -1- Cannot say *বলতে পারিনা* - -2- Decline to respond উত্তর দিতে নারাজ | EITHER RESPONDENT |  |  |
|  | OV_RATE | *Using any number from 0 to 10, where 0 is the worst facility possible and 10 is the best facility possible, what number would you use to rate this facility?*  0 থেকে 10 এর মধ্যে যেকোনো নাম্বার ব্যবহার করতে পারেন, 0 সম্ভাব্য খারাপ সূচক এবং 10 সম্ভাব্য সেরা সূচক, আপনাকে যদি এই কেন্দ্রকে নম্বর দিয়ে মুল্যায়ন করতে বলা হয় তবে আপনি এ কেন্দ্রকে 0 থেকে 10 এর মধ্যে কোন নম্বরটি প্রদান করবেন?  **Response codes**  Enter -1 for Cannot say বলতে পারিনা  Enter -2 for Decline to respond উত্তর দিতে নারাজ | EITHER RESPONDENT |  |  |
|  | OV_REC | *If a friend or family member needed health care, would you suggest to them that they come to this facility?*  *যদি আপনার কোন* বন্ধু বা পরিবারের সদস্যের স্বাস্থ্যের যত্নের প্রয়োজন হয়, তাহলে আপনি কি তাদেরকে এই সেবাকেন্দ্রে আসার পরামর্শ দিবেন?  **(SELECT ONE)** (একটি উত্তর নির্বাচন করুন)   - *1- No না* - *2-Yes, somewhat* হ্যাঁ, কিছুটা - *3- Yes, definitely* হ্যাঁ, অবশ্যই - --1- Don’t know জানিনা - -2- Decline to respond উত্তর দিতে নারাজ | EITHER RESPONDENT |  |  |
|  | OV_RETURN | *How likely are you to return to this facility for follow-up or if you needed medical attention for another illness in the future?*  আগামীতে যদি ফলো-আপ বা অন্য কোন ধরনের চিকিৎসার প্রয়োজন হয় তাহলে আপনার এই সেবাকেন্দ্রে পুনরায় আসার সম্ভাবনা কেমন?  **(SELECT ONE)** (একটি উত্তর নির্বাচন করুন)   - *1- Very unlikely* খুবই কম সম্ভাবনা - *2- Somewhat unlikely* কিছুটা কম সম্ভাবনা - *3- Unsure* অনিশ্চিত - *4- Somewhat likely* কিছুটা সম্ভাবনা - *5- Very likely* অত্যন্ত সম্ভবনা - -1- Cannot say বলতে পারি না - -2- Decline to respond উত্তর দিতে নারাজ | EITHER RESPONDENT |  |  |
|  | OV_RETURN_WHY | *Why are you unlikely to return to this facility in the future?*  আগামীতে এই সেবাকেন্দ্র থেকে সেবা নিতে পুনরায় আসার সম্ভাবনা কম কেন?  **Open prompt.** প্রম্পট করা যাবে  **(SELECT ALL THAT APPLY)** (একাধিক উত্তর গ্রহণযোগ্য)   - Took too long to get to the facility সেবা পেতে অনেক বেশি সময় লাগে - 2- Too expensive to get to the facility সেবা পেতে অনেক বেশী খরচ - 3- Bad experience with medical staff চিকিৎসা কর্মীদের সঙ্গে খারাপ অভিজ্ঞতা - 4- Bad experience with other staff অন্যান্য কর্মীদের সঙ্গে খারাপ অভিজ্ঞতা - 5-Treatment too expensive চিকিত্সা অত্যন্ত ব্যয়বহুল - 6-Poor medication availability অপর্যাপ্ত ঔষধ প্রাপ্যতা - 7-Medications too expensive ও ঔষধের দাম খুব বেশী - 8-Too much waiting time খুব বেশী সময় অপেক্ষা করতে হয় - 9-Poor staff availability অপ্রতুল স্বাস্থ্যকর্মী - 10- Facility environment is not good অপর্যাপ্ত কেন্দ্রের পরিবেশ ভাল না - 11- *Other reason,* please describe below অন্য কারণ, নীচে বর্ণনা করুন - --1- Don’t know জানিনা - -2- Decline to respond উত্তর দিতে নারাজ | Enable If OV_RETURN<4  EITHER RESPONDENT |  |  |
|  | OV_RETURN_WHY_OSP | What was the other reason why you are unlikely to return to this facility in the future?  আগামীতে আপনার এই সেবাকেন্দ্রে ফিরে আসার সম্ভাবনা কম কেন? ফিরে না আসার অন্যান্য কারণ কী? | Enable if Item 10 other selected in OV_RETURN_WHY |  |  |
|  |  | **DEMOGRAPHIC AND VISIT QUESTIONS FOR PATIENT** |  |  |  |
|  | RELIGION | What is your religion? আপনি কোন ধর্মাবলম্বী?  **(SELECT ONE)** (একটি উত্তর নির্বাচন করুন)   - 1=Islam ইসলাম - 2=Hinduism হিন্দু - 3=Christianity খ্রীস্টান - 4=Buddhism বৌদ্ধ - 5=Other (SPECIFY) অন্যান্য(বর্ণনা করুন) - -2=Decline to respond উত্তর দিতে নারাজ | EITHER RESPONDENT |  |  |
|  | RELIGION_OSP | *Please specify other religion:* | Enable if RELIGION = 5 |  |  |
|  | EDU_LEVEL | Upto what class have you studied? ENTER COMPLETED CLASS for literates. আপনি কোন পর্যন্ত লেখাপড়া করেছেন? যে ক্লাশ সম্পূর্ণ করেছে তা লিখুন  **(SELECT ONE)** (একটি উত্তর নির্বাচন করুন)   - 1 = Primary/Ebtadye (Class 1-5) (প্রাইমারি/এবতেদিয়া (১-৫ পর্যন্ত) - 2 = Secondary/ Dakhil (Class 6-10) (মাধ্যমিক/দাখিল (৬-১০ পর্যন্ত)) - 3 = Higher Secondary/Alim (Class 11-12) (উচ্চ মাধ্যমিক/আলিম (১১-১২ পর্যন্ত)) - 4 = Degree/Honors/Fazel (Class 13-15) (স্নাতক/সম্মান/ফাজেল (১৩-১৫ পর্যন্ত)) - 5 = Masters/Kamel (Class 16) (স্নাতকোত্তর/কামেল (১৬ পর্যন্ত)) - -1= Don’t know জানি না - -2= Decline to respond উত্তর দিতে নারাজ | EITHER RESPONDENT |  |  |
|  | HEALTH | In general, how would you rate (your/the patient’s) overall health status today  সাধারণভাবে, কিভাবে আজ (আপনার /রোগীর) সার্বিক স্বাস্থ্যের অবস্থা মুল্যায়ন করবেন?  **(SELECT ONE)** (একটি উত্তর নির্বাচন করুন)   - 1-Poor খুব খারাপ - 2- Fair মোটামুটি - 3- Good ভাল - 4- Very good খুব ভালো - 5- Excellent চমৎকার - -1- Cannot say বলতে পারি না - -2- Decline to respond উত্তর দিতে নারাজ |  |  |  |
|  | ATTN_REL | *What is your relationship to the patient?* রোগীর সাথে আপনার সম্পর্ক কি?  **Allow the attendant to identify relationship first; read options aloud if needed**  প্রথমে সহযোগীর এর সাথে রোগীর সম্পর্ক কি তা জানুন; প্রয়োজন হলে উত্তরগুলো পড়ে শোনান   - 1- Wife or Husband স্ত্রী অথবা স্বামী - 2- Son or daughter পুত্র বা কন্যা - 3- Son-in-law or daughter-in-law মেয়ের জামাই বা পুত্রবধু - 4- Grand child নাতি - 5- Parent পিতামাতা - 6- Parent-in-law শশুর-শাশুড়ী - 7- Brother or sister ভাই বা বোন - 8- Other relative অন্যান্য আত্মীয় - 10- Not related কোন সম্পর্ক নাই - --1- Don’t know জানি না - -2- Decline to respond উত্তর দিতে নারাজ | Enable-if: RESP_PT =0 |  |  |
|  | BIRTH | *Have you/the patient given birth in the last five (5) years?* আপনি/রোগী কি গত পাঁচ বছরের মধ্যে বাচ্চা জন্ম দিয়েছেন?  **(SELECT ONE )**(একটি উত্তর নির্বাচন করুন)   - 1- Yes হ্যাঁ - 0- No না - --1- Don’t know জানিনা - -2- Decline to *respond* উত্তর দিতে নারাজ | Enable if (SEX_PT=2) &((PT_AGE 16-49) OR (PT_AGE_CAT 2-5)) |  |  |
|  | B_WHERE | The most recent time you gave birth, where did you deliver the baby?  আপনি সম্প্রতি যে বাচ্চা প্রসব করেছেন, সে বাচ্চার প্রসব কোথায় হয়েছে?  **(SELECT ONE)** (একটি উত্তর নির্বাচন করুন)   - 1- At this facility এই সেবাকেন্দ্রে - 2- At another health facility অন্য স্বাস্থ্য সেবাকেন্দ্রে - 3- At home বাড়িতে - 4- Other reason, please describe: অন্য কারণ, দয়া করে বর্ণনা - --1- Don’t know জানিনা - -2- Decline to respond উত্তর দিতে নারাজ | Enable if BIRTH=1 |  |  |
|  | B_WHY | *Why did you not come to this facility to give birth?* বাচ্চার ডেলিভারীর জন্য এই সেবাকেন্দ্রে আসেননি কেন?  **Open ended prompt. উন্মুক্ত প্রশ্ন, প্রম্পট**  **(SELECT ALL THAT APPLY) (একাধিক উত্তর গ্রহনযোগ্য)**   - 1- Too far away খুব দূরে - 2- Transportation too expensive যাতায়াত খরচ বেশী - 3- Birth services too expensive ডেলিভারী সেবার খরচ বেশী - 4- The staff here are not respectful এখানকার কর্মীরা শ্রদ্ধাশীল না - 5- The medical staff here do not have good skills এখানকার চিকিৎসা কর্মীদের ভাল দক্ষতা নেই - 6- This facility does not have adequate equipment এই সেবাকেন্দ্রে পর্যাপ্ত সরঞ্জামাদি নেই - 7- Delivered near maternal home নিকটস্থ মায়ের বাড়িতে ডেলিভারী হয়েছে - 8- Clinic did not yet exist ক্লিনিকটি সেবাদানের উপযোগী ছিলনা - 9- *Other reason, please describe:* অন্য কারণ, দয়া করে বর্ণনা - --1- Don’t know- জানিনা - -2- Decline to respond উত্তর দিতে নারাজ | Enable if B_WHERE=2,3 OR 4 |  |  |
|  | B_WHY_OSP | *Please describe the other reason you did not come to this facility to give birth?*  *এই সেবাকেন্দ্রে ডেলিভারী সেবা নিতে কেন আসেননি তার অন্যান্য কারন দয়াকরে বর্ণনা করুন* | Enable if option 9 Other selected for B_WHY |  |  |
|  | COMMENT | *Please leave any comments you have about this particular interview, and report any problems or difficulties that arose.*  আপনি এই বিশেষ সাক্ষাত্কারে সম্পর্কে কোনো মন্তব্য, এবং যে কোন সমস্যা অভিযোগ বা অসুবিধার কথা দয়া করে বলুন |  |  |  |
|  | END_HH  END)MM | **Survey end time (HH/MM): সাক্ষাতকার শেষ করার সময় (ঘণ্টা/ মিনিট)** |  |  |  |
|  |  | *This is the end of the survey. Thank you very much for your time and participation and have a nice day.*  সাক্ষাৎকার এখানেই শেষ। আপনার সময় দেয়া এবং অংশগ্রহণের জন্য আপনাকে অনেক ধন্যবাদ এবং আপনার আজকের দিনটি সুন্দর কাটুক। |  |  |  |
